# Mere Nuisance or Growing Threat? The Physical and Economic Impact of High Tide Flooding on US Road Networks

**DOI:** 10.1061/(ASCE)IS.1943-555X.0000652

**Published:** 2021-12

**Authors:** Charles Fant, Jennifer M. Jacobs, Paul Chinowsky, William Sweet, Natalie Weiss, Jo E. Sias, Jeremy Martinich, James E. Neumann

**Affiliations:** 1 Senior Technical Consultant, Industrial Economics, Inc., 2067 Massachusetts Ave., Cambridge, MA 02140.; 2 Professor, Dept. of Civil and Environmental Engineering, Univ. of New Hampshire, 453 Morse Hall, Durham, NH 03824.; 3 President, Resilient Analytics, Inc., Resilient Analytics, 814 Trail Ridge Dr., Louisville, CO 80027; Professor, Civil, Environmental and Architectural Engineering, Univ. of Colorado, 314 UCB, Boulder, CO 80309.; 4 Oceanographer, National Oceanic and Atmospheric Administration, National Ocean Service, Center for Operational Oceanographic Products and Services, 1305 East-West Highway, Silver Spring, MD 20910-3281.; 5 Senior Research Analyst, Industrial Economics, Inc., 2067 Massachusetts Ave., Cambridge, MA 02140.; 6 Professor, Dept. of Civil and Environmental Engineering, Univ. of New Hampshire, 33 Academic Way, Kingsbury Hall, W183, Durham, NH 03824.; 7 Climate Scientist, US Environmental Protection Agency, 1200 Pennsylvania Ave., NW MC6207A, Washington, DC 20460.; 8 Principal, Industrial Economics, Inc., 2067 Massachusetts Ave., Cambridge, MA 02140.

## Abstract

High tide flooding (HTF) already affects traffic in many US coastal areas, but the issue will worsen significantly in the future. While studies show that large storm surge events threaten to be ever more costly, less damaging, but more frequent HTF events remain understudied and potentially carry a comparable economic impact. This study advances our understanding of the risks and impacts of HTF on vulnerable traffic corridors using hourly tide gauge water levels, sea-level rise projections, and link-level spatial analysis. It is the first study to estimate HTF economic impacts for varying levels of intervention, including reasonably anticipated driver-initiated rerouting and ancillary protection of adjacent property. The 2020 annual national-level costs of $1.3 to $1.5 billion will increase to $28 to $37 billion in 2050 and $220 to $260 billion in 2100 for medium to high greenhouse gas (GHG) emissions scenarios, respectively. Total costs over the century are $1.0 to $1.3 trillion (discounted 3%). Additional cost-effective protection by building sea walls or raising road surfaces could significantly reduce 2100 costs to $61 to $78 billion, but there remain many barriers to adopting least-cost adaptation decisions, and these gains may only be realized with careful planning and information sharing.

## Introduction

Rising sea levels threaten to change the nature of coastal flooding events by shifting vulnerable areas further inland, exposing infrastructure that was believed to be resilient to these hazards when it was first designed and constructed. The shift is observable now in many regions of the world (IPCC 2012; Emanuel 2013; Hinkel et al. 2014; Sundermann et al. 2014) and has challenged the way we think about infrastructure design and planning, especially for civil infrastructure (e.g., roads, structures, and pipelines), which are exposed to the environment and are built with the expectation of multiple decades of useful life (Qiao et al. 2020). Considering these expected changes, observed environmental conditions alone are often not sufficient for design and planning—future projections of these conditions are essential in many cases. Planning around flood events is no different, especially for coastal infrastructure in which rising sea levels will exacerbate storm surge and fluvial flooding by raising the flood depth and expanding threats to newly vulnerable areas (Dinan 2017; Neumann et al. 2015b; Hallegatte et al. 2013).

Many studies have evaluated low probability, high damage events (e.g., around 10- to 100-year return periods) for coastal infrastructures, such as storm surges (Hallegatte et al. 2011; Klima et al. 2012; Lin and Shullman 2017; Neumann et al. 2015b; Diaz 2016; Wahl et al. 2017; Dinan 2017), but there has been less focus on high probability, low damage high tide flood (HTF) events (sometimes referred to as minor, nuisance, or sunny day flooding). Historically, HTF events have not caused great damage to infrastructure or posed risks to public safety, at least not to the extent of more extreme events. But due to sea-level rise, HTF is increasingly putting more infrastructure at risk of flooding, with cumulative impacts mounting. Impacts include delays in traffic (Jacobs et al. 2018a; Suarez et al. 2005), declining real estate values (Nabangchang et al. 2015), or damage to low-lying or underground infrastructure, such as storm sewers (Cherqui et al. 2015). Although HTF causes less damage per event than extreme events, expected annual damage may be larger over time than extreme events in some cases because they happen more often and in more locations (Moftakhari et al. 2017). However, damage associated with HTF has been the subject of less monitoring and research to date, and links between specific event characteristics, such as depth and the associated impacts, are not well understood (Jacobs et al. 2018a; Moftakhari et al. 2018).

The frequency of HTF varies through time with some noticeable intraannual-to-decadal signals, driven by a variety of oceanatmosphere dynamics and physical phenomena, such as the 18.6-year lunar nodal cycle or modes of the El Nino Southern Oscillation (ENSO) (Sweet and Park 2014; Sweet et al. 2018; Thompson et al. 2019). Aside from these oscillations, HTF occurrence and depth are also shifting over time as a result of regional sea-level dynamics, subsidence, and global sea-level rise (SLR) (Sweet and Park 2014; Sweet et al. 2018). High tide flooding has already caused noticeably increased impacts in coastal cities such as Annapolis, MD, Norfolk, VA, Miami Beach, FL (Sweet et al. 2019), and, to a lesser extent, San Francisco (Moftakhari et al. 2017). Analysis of hourly tide gauge measurements between 2000 and 2015 indicates that annual frequencies of HTF have increased by about 125% in the Southeast Atlantic and 75% for both the Northeast Atlantic and Western Gulf (Sweet et al. 2018). Using the intermediate sea level rise projection (global rise of 1 m by 2100), these events are expected to increase from 1.3 days/year in 2000 to 85 days/year in the Southeast, 3.4 to 130 days/year in the Northeast, and 1.4 to 185 days/year in the Western Gulf by 2050 (Sweet et al. 2018). The Eastern Gulf and Pacific Coast are expected to experience less dramatic shifts in tidal flooding events.

Increases in tidal flooding events are likely to have long-lasting impacts on traffic and roadway maintenance in coastal regions. Roads are already vulnerable to changes in temperature and precipitation, causing degradation, more frequent maintenance, or the road surface to be redesigned to be resilient to the projected climatic conditions (Jacobs et al. 2018b; Underwood et al. 2017; Neumann et al. 2015a; Mallick et al. 2018). Neumann et al. (2021) estimate that direct and indirect costs to noncoastal road infrastructure caused by climate change would be roughly $90–$140 billion/year by 2050 in the contiguous United States (CONUS) under a no-adaptation scenario but could be reduced by over 90%, to $8 billion/year, with proactive adaptation. The study points out that most of the avoidable costs—i.e., the difference between no adaptation and proactive adaptation—are from traffic delays and damage to vehicles, placing the cost burden on drivers. Batouli and Mostavi (2018) developed a model of multiagent adaptive responses to changes in roadway maintenance costs from HTF driven by sea-level rise and precipitation projections and applied the model to a road network in Miami. Their study finds that with adaptation, average network lifecycle costs would be roughly $3.7–$4.8 million per year compared to $15–$17 million without adaptation.

To date, little is known about the impacts of HTF on CONUS road infrastructure over a long planning horizon (through 2100) when considering regional sea level rise scenarios (Sweet et al. 2017). Jacobs et al. (2018a) combined hourly historical water levels, a public road infrastructure database, and fine-scale flood mapping to estimate HTF impacts on traffic delays (in vehicle-hours) for east coast states. Their study introduced a unique procedure that defines road segments at intersections for the purpose of estimating traffic delay risks to HTF. Jacobs et al. (2018a) found that delays would increase substantially along the East Coast, reaching 1.2 billion vehicle-hours by 2060 and 3.4 billion by 2100, whereas all delays combined in the US currently are roughly 100 million vehicle-hours. While Jacobs et al. (2018a) indicate HTF will result in significant regional impacts, to date, no national-scale study has quantified the economic impact of HTF and sea-level rise on the coastal road network nor considered multiple levels of adaptation potential that could be pursued to mitigate these effects.

In this study, we build on Jacobs et al.’s (2018a) methods by expanding the evaluation region to include the Gulf and West Coasts, estimating the economic costs of traffic delays, and exploring various adaptation options that could make the road network more resilient. Specifically, the contributions of this study to the literature are the following. We evaluate indirect adaptation options to alleviate these traffic delay costs by (1) considering road network redundancy by using a detour effectiveness measure; and (2) accounting for roads that would be protected by actions to protect property from storm surge and/or SLR given a least-cost decision by property owners. In addition, we explore options to protect roads directly by applying a screening-level least cost decision and cost evaluation for two adaptation options: raising the road profile and building a sea wall. Finally, we consider changes to maintenance costs that arise from more frequent inundation for a single road segment to illustrate how adaptation costs to prevent direct damage compare to delay costs.

This analysis is being conducted as part of the ongoing Climate Change Impacts and Risk Analysis (CIRA) project, which uses a consistent analytical framework of socioeconomic scenarios and climate projections to estimate and compare economic impacts across multiple sectors and regions (Martinich and Crimmins 2019; EPA 2017).

## Methods

**Fig. 1.**
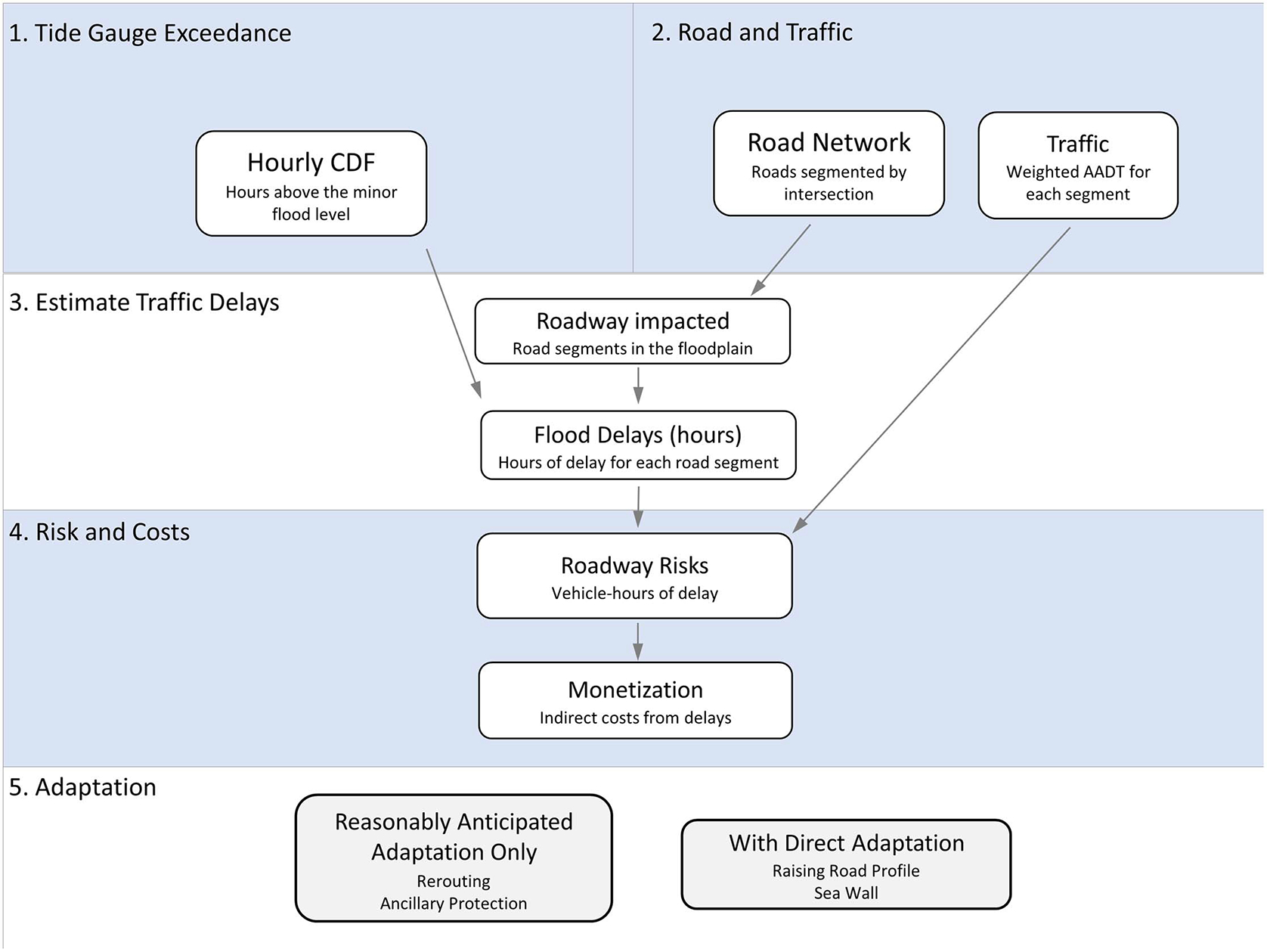
Model framework.

Following the framework outlined by Jacobs et al. (2018a), risks of traffic delays are estimated for CONUS following the first four steps shown in Fig. 1. In summary, the hourly empirical cumulative density function (CDF) of tide gauge water levels is determined (#1). The road network is segmented by intersections or ramps, and traffic data are assigned to each segment (#2). These datasets are used with the flood plain to identify vulnerable roads and flood duration (#3). Roadway risk, i.e., vehicle hours of delay, is calculated as the product of the flood duration and traffic. The risk is then monetized using hourly rates for passenger and freight truck traffic delays, and costs of direct damage to the road surface are also explored in a sensitivity analysis (#4). Two types of adaptations are simulated (#5): reasonably anticipated adaptation, which includes driver-initiated rerouting and ancillary protection from actions to protect property, and direct adaptation, in which, in addition to reasonably anticipated adaptation, actions are taken to alleviate delays by raising the road profile or building hard structures, such as sea walls.

We now go into further detail. A full list of the data used in this study and the sources can be found in Table S1 of the Supplemental Materials.

### High Tide Exceedance Projections and Flood Extent

Hourly water levels from tide gauge stations were obtained from NOAA’s Center for Operational Oceanographic Products and Services (NOS 2019). The coverage of tide gauges with locally derived nuisance levels is poor along the Pacific and Gulf coasts. To account for this, we employ recently developed *minor* flood levels as thresholds (Sweet et al. 2018) instead of the nuisance levels, which apply a consistent approach and definition across gauges and allow the use of any gauge with sufficient hourly records.

Using 19-years of hourly water levels spanning from 1999 to 2017, flooded hours were estimated using the approach described in Jacobs et al. (2018a). First, the hourly record of water levels is detrended and brought to the baseline year 2000. Using an empirical CDF, hours above the HTF threshold level provides the number of hours flooded for the baseline. These flooded hours are then estimated over time by adding the differences from the sea level in 2000 to the six local sea-level rise projections from Sweet et al. (2017) to the water level CDF. This approach is limited to tide gauge measurements and does not consider situations in which flooding is intensified or induced by precipitation.

We link the 83 tide gauges in CONUS with sufficient hourly records with the road network at the county level. Each of the 302 coastal counties was assigned the tide gauge closest to the midpoint of the shoreline of the state, followed by manually checking each county for a clear hydrologic connection and reassigning if necessary. Note that the 83 tide gauges may not be an accurate representation of HTF for all coastal roads, especially roads further from the assigned tide gauge.

NOAA’s flood maps derived from 30-cm resolution LIDAR digital elevation data using a modified bathtub approach are used to delineate the flood extent at the specified levels (*reference to dataset*). Road segments within the NOAA flood extent are designated as road segments vulnerable to HTF. Using the NOAA flood map extents limits the analysis to the roads that are currently vulnerable to HTF. With rising sea levels, flood extents will increase, and additional roads will become vulnerable that are not currently vulnerable. While this effect is not included in the analysis, we apply a first-order approximation of the effect of flood plain expansion with the SLR using the National Coastal Property Model (NCPM: Neumann et al. 2010, 2015b; Lorie et al. 2020), which uses a topography composed of 150-m grids. This approach is not as precise as the 30-cm LIDAR approach from NOAA and may not capture road surface elevations.

### Road Segments and Traffic

Our study adapted the geospatial methods outlined in Jacobs et al. (2018a) to process 2016 Highway Performance Monitoring System (HPMS 2016) roads data into consistent road segments across states and determine which are vulnerable to tidal flooding. We used ArcMap 10.4 and ET Geowizards to process raw HPMS road segments into continuous road segments. We processed the HPMS data depending on the road functional class; Functional classes 1 and 2 (interstates and principal arterial freeways and expressways, respectively) segments were defined as continuous segments between on and off-ramp intersections, and Functional classes 3 to 7 (other principal arterials, minor arterial, major collector, minor collector, and local, respectively) segments were defined as continuous road segments between intersections. We made a few modifications to the Jacobs et al. (2018a) approach, including an augmentation to account for incorrect bridge locations, which are explained in detail in Section S2 of the Supplemental Materials. The quality of the HPMS dataset varies by state, and Section S2 also provides a detailed account of the variations with examples in Fig. S1.

We calculate vehicle miles traveled (VMT) for each original road segment (Eq. 1) and reassigned these to the newly defined segments using a weighted average annual daily traffic (AADT) value

(1) $${\text{VMT}}_{i}={\text{AADT}}_{i}\times {\;\text{Length}}_{i}$$

where VMT*i* = vehicle miles traveled for each segment *i*; and Length = segment length for segment *i*. The weighted AADT is calculated by summing the VMT for each road segment in the newly defined segment and dividing the total VMT by the total length of the newly defined segment.

We expect that socioeconomic changes over the century will have an impact on traffic. To account for these changes, we apply population and economic projections to baseline AADT, in which the number of passenger vehicles grows linearly with changes in population, and the number of heavy vehicles, which primarily transport goods, grows linearly with projections in the gross domestic product (GDP). Consistent with other CIRA sectoral analyses, we use population projections at the county level from the Integrated Climate and Land Use Scenarios version 2 (ICLUSv2) model (Bierwagen et al. 2010; EPA 2017) under the median variant projection of the United Nations (2015). The projected population changes in ICLUS are based on assumptions of medium levels of fertility, mortality, and migration (O’Neill et al. 2014). GDP projections are based on a combination of data from the 2016 Annual Energy Outlook (USEIA 2016) and a run of the Emissions Prediction and Policy Analysis (EPPA, version 6) model (Chen et al. 2015). Based on these projections, the population of coastal counties increases from 115 million in 2016 to 173 million in 2100, a ratio of 1.51, while the national GDP is 4.3 times higher in 2100 than 2016. Note that based on the HPMS dataset for 2016, about 6.2% of traffic is from heavy vehicles across all road segments in the minor flood plain.

### Delays and Delay Costs

Traffic delays are determined using the product of the lengthweighted AADT and the flooded hours from the exceedance projections, in units of vehicle-hours, following Jacobs et al. (2018a). The indirect costs of traffic delays are estimated using hourly rates for both passengers and freight vehicles. To quantify the unit cost of delay for passenger vehicle-hours, the value of travel time savings (VTTS) estimates from the US Department of Transportation’s 2016 guidance—$20.40 ($2,015 per person-hour)—are used. The average occupancy of passenger vehicles is estimated from the 2017 National Household Travel Survey to be 1.67 (FHWA 2013).

Freight traffic, which consists of both combination truck AADT and single-unit truck and bus AADT, are both available in the HPMS dataset. The National Cooperative Highway Research Program (NCHRP) inputs to their truck freight reliability valuation model (NCHRP 2016) are used to quantify the hourly cost of delay for freight vehicles. These costs include $65 per delay hour for operating and maintenance costs (including fuel, truck/trailer lease or purchase payments, repair and maintenance, and driver wages and benefits) and $35 for cargo-related supply chain costs, for a total of $100 ($2,015) per delay hour per truck.

### Road Maintenance Costs

Flooding is a natural disaster that can have a severe impact on roads and has caused significant economic loss to road infrastructure worldwide. Most impact studies focus on roads that have been severely damaged or washed away due to erosion. However, even when a flood does not completely wash away the roadway, the structural capacity of the pavement can be significantly reduced due to the inundation of unbound materials. The rapid damage under traffic in these flood conditions has only recently begun to receive consideration in traditional pavement design and management. In addition to the indirect delay costs, direct damage to roads from these flooding events is considered. The details of the approach are described in Section S3 of the Supplemental Materials.

Because these costs are highly dependent on local conditions, including construction materials, permeability, and hydrology, estimating these direct costs for CONUS is beyond the scope of this study. For this reason, we evaluate these costs for a single road segment near Hilton Head, SC, to illustrate the procedure and compare these direct costs to the indirect delay costs.

### Reasonably Anticipated Adaptations

Reasonably anticipated adaptations are actions that reduce delays and are likely to occur independently of delays caused by high tide bypass of a flooded road and ancillary protection in which high tide flooding is prevented using protective strategies, such as sea walls and beach nourishment.

### Alternative Routes

If a driver encounters a flooded, impassable road, it is likely that (1) the driver will attempt an alternative route, if it exists, using GPS or smartphone technology, or (2) an official detour will be in place set up by local transportation departments or other agencies. These alternative routes take advantage of route redundancy in the road network, the effectiveness of which varies by location. The effectiveness of these detours also depends on other factors, including the start and end of the driver’s trip, the redundancy of the road network and its effectiveness in providing uncongested alternative routes for the specific trip, and the importance of the trip to the driver (if there are delays, the driver may choose to abandon the trip), among others. These factors are complex and would require a detailed trip network analysis, which is too complex for a national scale study, but could be further informed by ongoing work in this area (e.g., Kurth et al. 2019).

For this study, we account for route redundancy, using a slightly modified version of the traffic intensity indicator (Cam. Sys. 2005) to reduce delays where the road network is extensive and more likely to provide reasonable alternative routes. The details of this approach are described in Section S4 of the Supplemental Materials.

This indicator approach does not consider the additional time it takes for the driver to reroute around that which is impacted or the value of delays associated with drivers taking the alternate route. While these delays could be substantial at a national scale, we leave this for future research or smaller-scale (e.g., neighborhood) analysis in which the necessary data can be more readily accessible.

### Ancillary Protection

In certain cases, these flooded roads are in or near valuable development. Actions to protect property, such as sea walls or beach nourishment, may also protect the road itself if the road is further inland. In addition to road redundancy, we also consider avoided flooding and delays that result from actions that are taken to protect coastal property. The NCPM estimates damage from storm surge and inundation losses and compares these to various adaptation options, including sea walls and beach nourishment, indicating the areas protected behind these improvements. Because these adaptation strategies protect against storm surge water levels around the 100-year event, they are well equipped to prevent roadway flooding during HTF events.

## Direct Adaptation

The direct adaptation method considers the alleviation of HTF-induced traffic delays through the implementation of adaptation strategies. While there are many conceivable ways to adapt to HTF, we model two well-established options: (1) build a sea wall to hold back the floodwater; and (2) raise the road profile above the effective threshold.

For each road, the decision to adapt using one of these two options depends on the ratio of benefits to costs. Lorie et al. (2020), among others, point out that in many cases, the benefits (*B*) need to be significantly higher than the costs (*C*) to trigger action. We follow Lorie et al. (2020) by running multiple scenarios varying this *B*/*C* threshold, S, where adaptation is built when the following (Eq. 2) is true

(2) $$\frac{B}{C}\geq S$$

We develop scenarios using S-values of 1, 2, 4, and 10. Benefits, *B*, are the avoided traffic delay costs for 10 years, an assumed planning horizon to trigger protection, discounted by 3%. The cost of a sea wall is related to the square of its height, essentially a triangle with a wide base, following previous studies (Yohe et al. 1999; Mendelsohn et al. 2019; among others). The cost of raising the road profile is composed of the material and labor costs of adding base thickness and embankment. Elevating roads requires additional widths for the embankment, and obstacles or development near roads often make this infeasible. We account for this using available information in the HPMS dataset, which excludes roughly 13.7% of the road segments but 48.5% of the total traffic because these are often on high-trafficked roads in urban areas. Other constraints such as soil types that lack sufficient structural stability are not addressed. Details of this and the adaptation costs are provided in Section S5 of the Supplemental Materials.

While the adaptation costs include estimates of material, labor, and construction delays, actual costs will include additional factors—for example, costs associated with management, design, easements, or land acquisition (see Table S2 of the Supplemental Materials for a longer list). In addition, our framework implies that protection will be built without design or construction errors or schedule delays due to sociopolitical or budgetary issues. While construction of either of these protection types would divert floodwaters away from the road, flooding may occur elsewhere as a result, and additional costs would be incurred. Furthermore, these protections may have environmental impacts, such as preventing wetland migration.

## Results

Our method results in six sequential categories of results, which build upon the prior step:

- Roadway vulnerability, identifying the extent of roads that could be vulnerable to HTFs;
- Road flood risk, assessing the intersection of vulnerable roads with the timing and depth of flood hazards, as well as the intensity of traffic, ultimately denominated in hours of delay;
- Reasonably anticipated adaptations, which reduce expected delays by incorporating the effect of risk mitigation;
- Indirect costs, in which delay is valued, considering differential values for freight and passenger vehicle delay times;
- Direct costs of flooding on pavement integrity (a case study); and
- The costs of direct adaptation simulations.

### Roadway Vulnerability

In CONUS, approximately 30,000 segments and about 12,000 miles of roads are within the minor flood extent. The majority of these roads are in Functional classes 6 and 7, which represent 70% of the total segments and 51% of the total miles. Functional classes 1 and 2 are the least common, with 1,200 miles and less than 700 segments. The total AADT for all segments that intersect the flood plain for the 23 coastal states, which excludes Alaska and Hawaii, is 119 million vehicles per day. California, Texas, and Massachusetts have the most traffic vulnerable to HTF and make up over half of the total vulnerable traffic, presenting 18%, 18%, and 16% of the total, respectively (Fig. S2 in the Supplemental Materials).

As mentioned in the “High Tide Exceedance Projections and Flood Extent” section, we use the NCPM to allow the flood plain to migrate with SLR and evaluate the total vulnerable traffic over the century for the intermediate-low (50 cm) and intermediate (100 cm) scenarios in order to gauge the effect of flood plain migration to new road segments. We find that the total vulnerable traffic increases by 25% and 48% in 2050 and 66% and 120% in 2100 for the intermediate-low and intermediate scenarios, respectively. This flood plain migration is not included in the remainder of the results.

### Roadway Flood Risks

**Fig. 2.**
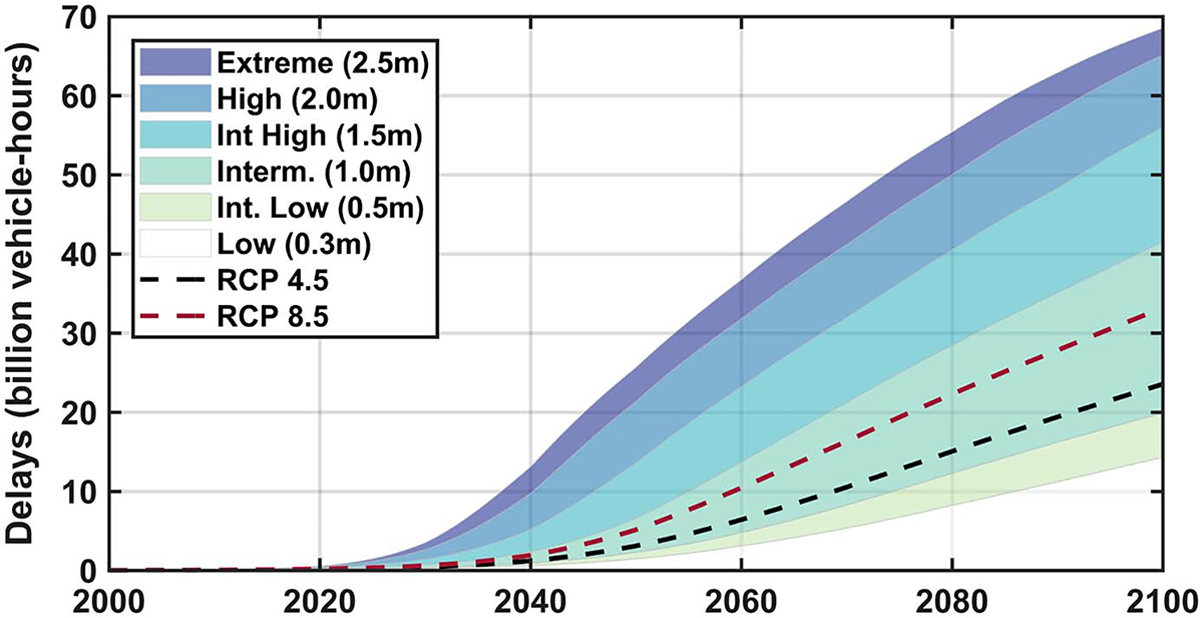
Roadway flood risk for the six sea level rise scenarios for all of CONUS.

Changes in flood duration from 2000 to 2100 are estimated by applying local SLR scenarios to the tide gauge water levels. Roadway flood risks are calculated as the product of duration and segment traffic. Fig. 2 shows the total flood risk for all road segments in the flood plain in CONUS. In 2000, these delays were slightly less than 50 million vehicle-hours. By 2020, the delays range from 130 to 570 million vehicle-hours, by 2050 from 1.5 to over 26 billion vehicle-hours, and by 2100 from 14 to 69 billion vehicle-hours. In many cases, especially for the higher sea level rise scenarios (e.g., extreme), segments are inundated even during low tides. Using the probability of exceedance (USGCRP 2017), we apply weights to the six sea-level rise uncertainty scenarios to approximate the effect of two greenhouse gas emissions scenarios on the net expectation of SLR, consistent with radiative forcing associated with the representative concentration pathways (RCP) 4.5 (lower emissions) and 8.5 (higher emissions). The results for RCPs 4.5 and 8.5 both fall within the range of the intermediate-low and the intermediate SLR scenarios.

### Reasonably Anticipated Adaptations

**Fig. 3.**
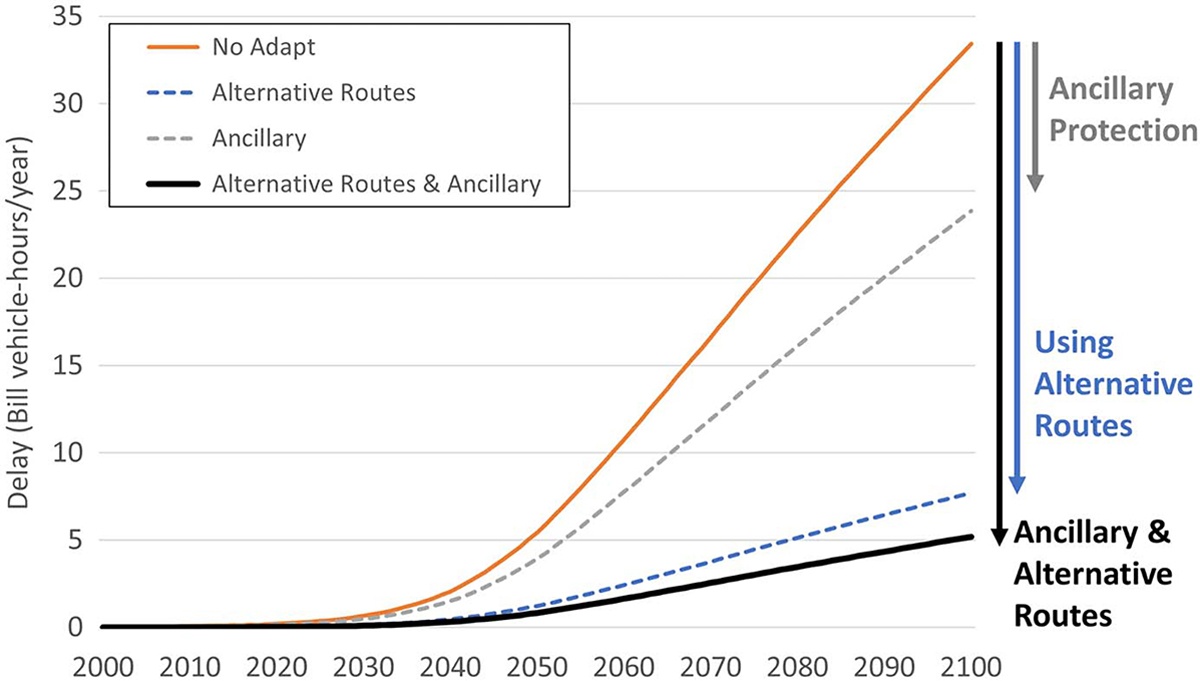
Roadway flood risks with reasonably anticipated adaptation for RCP 8.5 in CONUS

Incorporating alternative route efficiency and ancillary protection substantially reduce these delays (see Fig. 3 for RCP 8.5). For both RCPs, ancillary protection reduces delays by 28% as compared to delays without ancillary protection in 2050 and by 29% in 2090. Alternative routes reduce delays by about 77%, independent of RCP or time.

Although these reasonably anticipated adaptations always reduce delays, the effects vary by place. For example, ancillary protection reduces delays in New York by 50%, higher than the national average because much of the higher property value coastal region in New York state is expected to yield ancillary protection for nearby, and otherwise vulnerable, roadways. By contrast, alternative routes only reduce delays by 46%, lower than the national average due to higher road congestion associated with population and traffic density. These reductions in delays from rerouting are in line with other city-scale assessments in the US, such as those by Kurth et al. (2019).

**Fig. 4.**
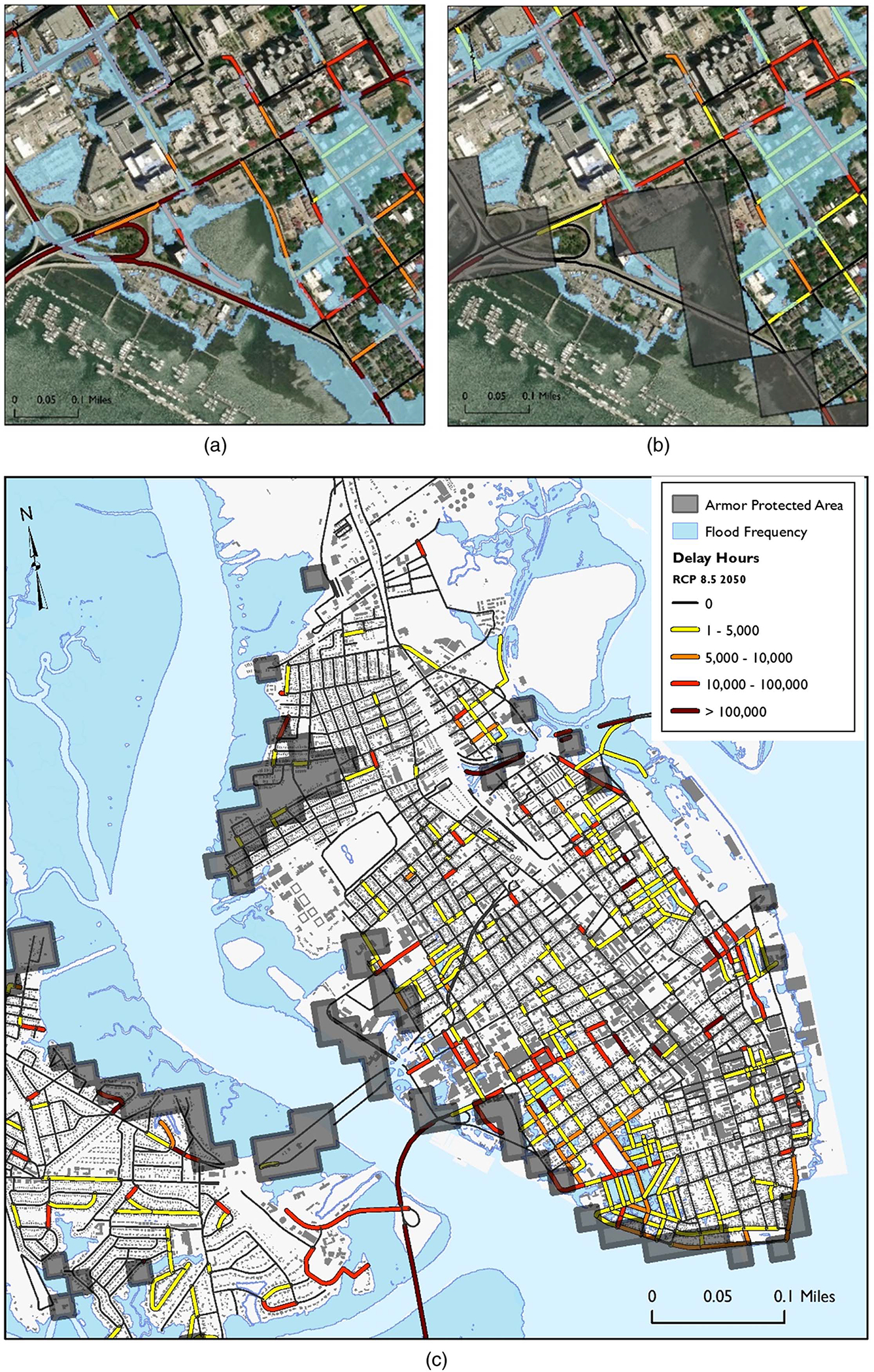
Maps of downtown Charleston in 2050 with closeup maps of (a) delays without adaptation; (b) with reasonably anticipated adaptation; and (c) map of downtown Charleston with reasonably anticipated adaptation. (Map data from Esri, Maxar, GeoEye, Earthstar Geographics, CNES/Airbus DS, USDA, USGS, AeroGRID, IGN, and the GIS User Community.)

In Charleston County, SC, ancillary protection reduces delays by 47% for RCP 8.5 by 2050, and alternative routes reduce delays further by about 42%, bringing delays to 11% of the original values without these factors considered. Fig. 4 shows maps of downtown Charleston with and without reasonably anticipated adaptation. As shown in the differences between Figs. 4(a and b), reasonably anticipated adaptation reduces delays considerably. In Fig. 4(a), many road segments experience delays of over 150,000 vehicle hours. Ancillary protection [grey blocks in Figs. 4(b and c)] protects many of these segments, reducing delays to zero.

At low flood depths, drivers may be able to pass through an overtopped roadway with only minor speed reductions. A 13-cm water depth reaches the undercarriage of most vehicles (Gattis et al. 2010; Shand et al. 2011), although many trucks or SUVs have higher clearance. Absent of high-resolution and precise road surface elevations, the actual flood depth is unknown. In addition, our analysis relies on the flood extent provided by NOAA at the minor level, as discussed in “High Tide Exceedance Projections and Flood Extent.” This method designates all roads below the minor level; however, the actual surface may be lower than that level such that flooding occurs more often. For these reasons, the effective threshold in which the road is no longer drivable (set at the minor level in this study) is an important and uncertain parameter. Fig. S3 in the Supplemental Materials shows costs at these levels. In sum, increasing or decreasing the effective depth shifts delay results by about 0.9% per centimeter.

### Indirect Costs

Indirect costs of delays combined with standard value-of-time methods that reflect labor costs and vehicle usage were used to monetize delays. Costs vary by functional class, with about 39% in Functional classes 1 and 2, 57% in Functional classes 3–5, and only 4% in Functional classes 6–7. Across CONUS, annual costs are $1.3 and $1.5 billion in 2020 for RCP 4.5 and RCP 8.5, respectively. These annual costs increase $28 and $37 billion in 2050 and $220 and $260 billion in 2100 for RCP 4.5 and RCP 8.5, respectively. Using a discount rate of 3%, total costs over the century are $1.0 trillion for RCP 4.5 and $1.3 trillion for RCP 8.5. Roughly 40% of these costs are due to socioeconomic growth, meaning that costs would be about 40% lower if traffic rates were static at 2016 levels.

**Fig. 5.**
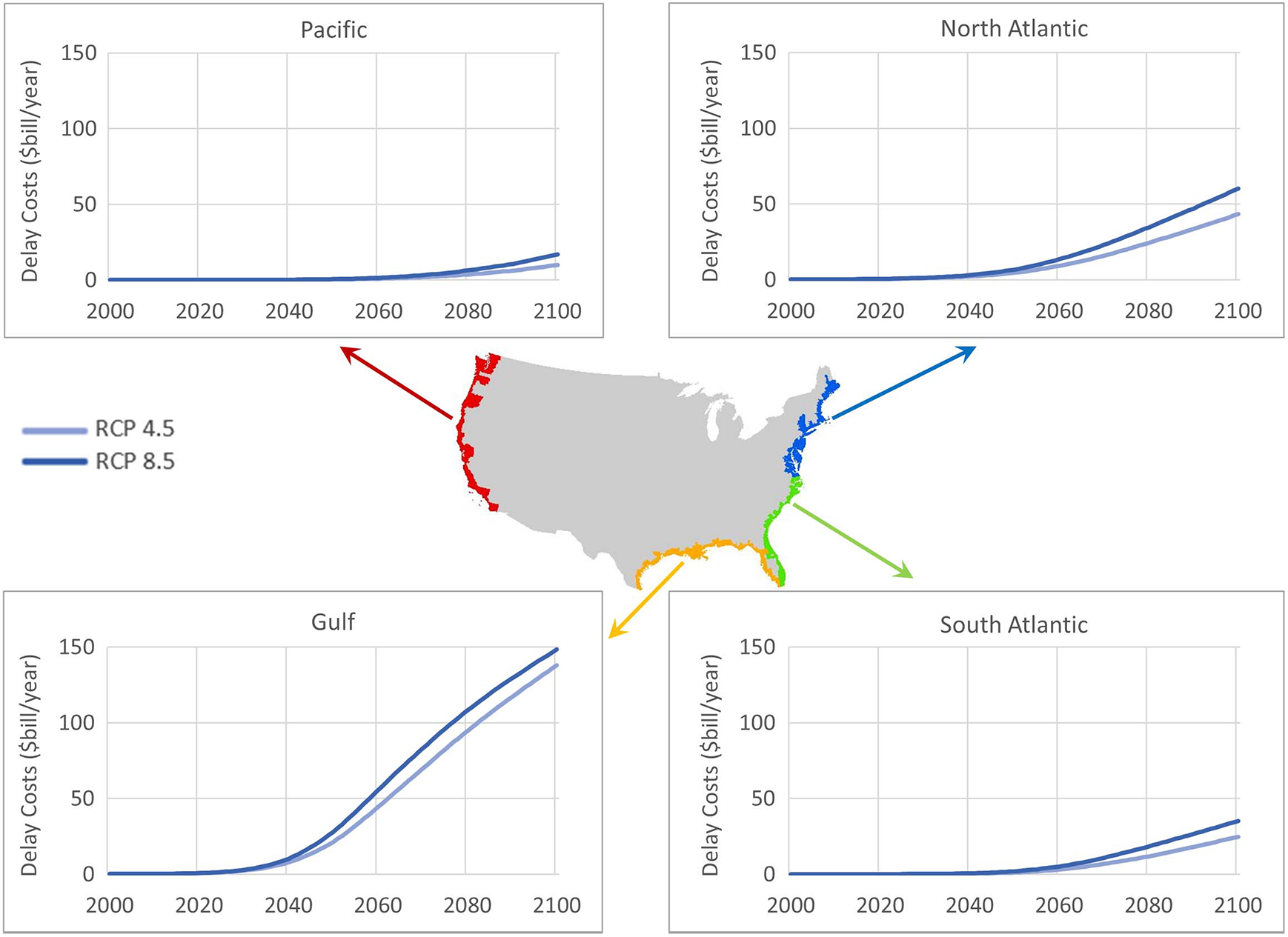
Regional delay costs for RCP 4.5 and RCP 8.5.

Costs for four coastal regions—the Pacific, Gulf, North Atlantic, and South Atlantic—are shown in Fig. 5 for delays with reasonably anticipated adaptation (also, see Table S4 for annual cost values). The differences between RCP 4.5 and RCP 8.5 are relatively minor until after 2050 for all regions except the Gulf Coast, although these do not account for the entire range of uncertainty, which is higher for RCP 8.5 due to uncertainties related to the potential disintegration of the Antarctic ice sheets and subsequent effects on sea levels.

Costs are noticeably higher in the Gulf, where subsidence and accelerated sea-level rise rates cause significant delays, primarily along the Texas and Louisiana coasts. Around 2070, costs start to decelerate slightly because many of the roads are inundated even at low tides, although it is important to restate the limitation regarding static flood extents described in the section “High Tide Exceedance Projections and Flood Extent.” Comparatively, delay costs in the Pacific are relatively lower from lower sea-level rise rates, with most of the costs concentrated in a few counties around San Francisco and Los Angeles where five counties account for 80% of the costs by 2100 for RCP 8.5. In the South Atlantic, costs are dominated by results for Miami Dade County, which accounts for over half of the total costs for the region by 2100—over $19 billion/year—for RCP 8.5. Because the North Atlantic has greater topographic relief, generally, as compared to the South Atlantic, it has relatively less inundated land area overall. However, the North Atlantic traffic volume is significantly heavier. For this reason, we find significant delay costs by 2100 for the Boston ($10 billion/year) and New York ($7.2 billion/year) metropolitan areas.

**Fig. 6.**
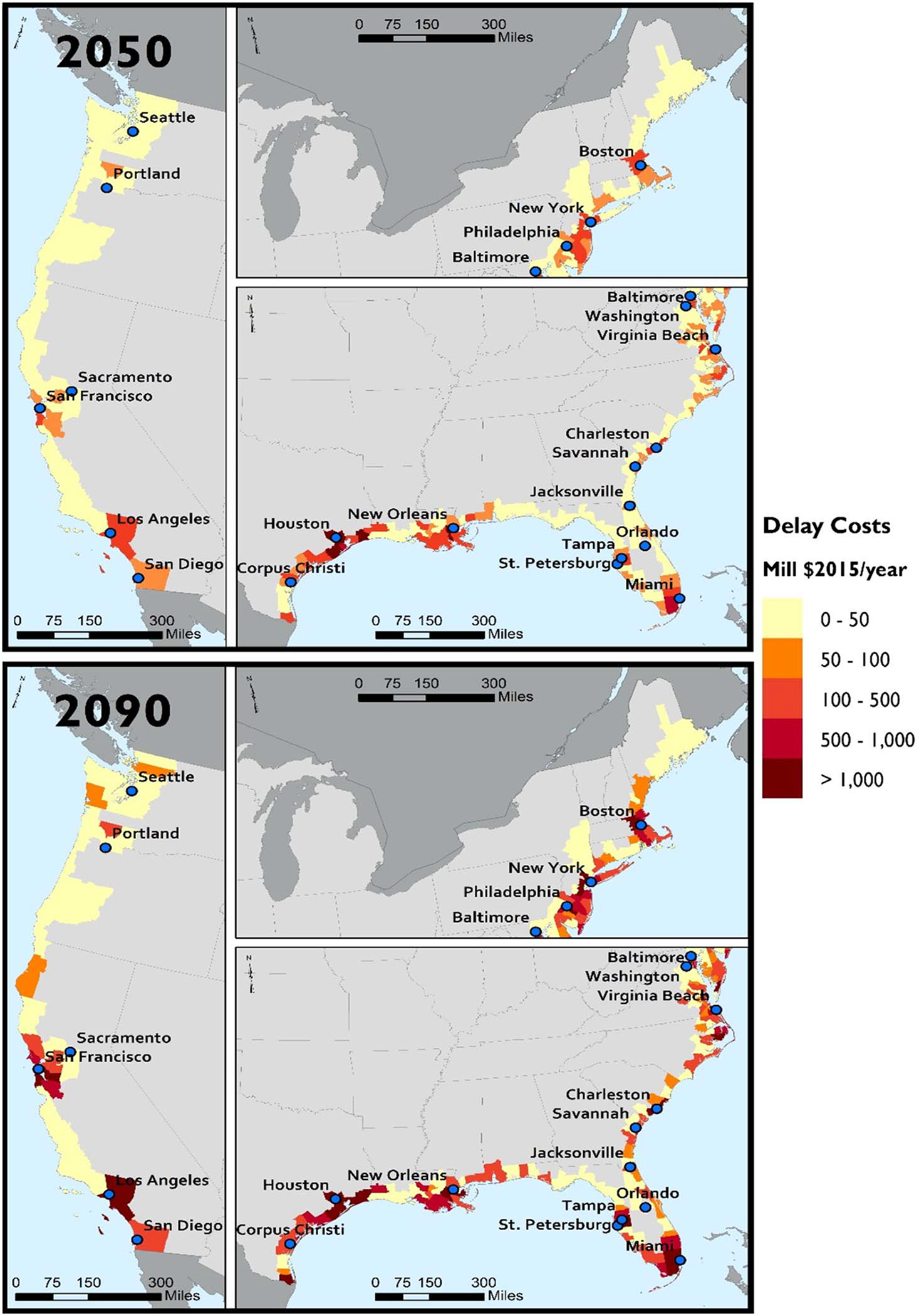
Map of delay costs (in millions of 2015 USD/year) by county for reasonably anticipated adaptation and RCP 8.5.

RCP 8.5 delay costs for 2050 and 2090 by coastal county are shown in Fig. 6. By 2050, delay costs for most counties are less than $50 million. Delay costs for RCP 4.5 are similar and shown in Fig. S4 of the Supplemental Materials. The area with the highest delay costs is near New Orleans and eastern Texas, around Houston and Galveston. In fact, New Orleans (Orleans and Jefferson Counties, LA) accounts for $12 billion of the total $37 billion/year by 2050. Similarly, five counties in and around Houston also account for about $12 billion/year in 2050. Near the end of the century, costs escalate quickly. New Orleans reaches $59 billion/year, and the Houston area reaches $65 billion/year. New York and New Jersey counties account for over $26 billion/year and Massachusetts $13 billion/year by 2100.

### Direct Repair Costs

HTF is likely to cause damage to the road structure. To understand the potential magnitude of these costs in relation to indirect costs from delays, we evaluate the impacts to a road segment near Hilton Head, SC, on Route 21N. Costs are in terms of additional pavement (asphalt surface layer thickness) that would be required to maintain the same structural capacity and useful life without high tide flooding. These are based on an asphalt replacement cost of $100/ton, which includes about $85 for materials and $15 for labor (Gordian 2019). Additional delays during repair are not considered. Also, realistically, the thickness would be rounded up to the next half inch, but these were not rounded for the sake of the comparison with the delay costs. Table 1 shows the mean characteristics per year of the high tide flood events by 2050. The average duration of these events is about 2 h, and we assume that normal traffic levels occur after the flood has receded from the road. The analysis shows that direct repair costs are roughly 0.8% and 1.3% of the indirect costs, with reasonably anticipated adaptation, per tenth of a mile for the intermediate low and intermediate sea level rise scenarios, respectively, by 2050. Note that these costs assume the road is regularly maintained and does not consider delays caused by deteriorating road conditions, e.g., drivers slowing down to avoid ruts or potholes.

### Direct Adaptation

The study identified when the benefits are equal to or above protection costs and considered when to adapt in response to traffic delay costs by either building a sea wall or raising the profile using various cost-to-benefit ratios. The results show that direct adaptation is more cost-effective than repeated delays. Even in the first 20 years of the simulation, which is essentially a hindcast, adapting is more cost-effective than incurring HTF effects. By 2050, between 52% and 63% (range is from the six SLR scenarios) of the total vulnerable traffic is protected by the direct adaptation for an *S*-value of 1, in which protection is built when the benefits are equal to or above protection costs. Even with an *S*-value of 4, when benefits are four times the protection costs, between 29% and 42% of total traffic is protected in 2020. By 2050, 60%–69% of the traffic is protected, and by 2100, 68%–74% of the traffic is protected with an *S*-value of 4. Table 2 shows the portion of total segments and total traffic protected by the direct adaptation for both protection types for the 100-cm (intermediate) SLR scenario. Most of the road segments are protected by raising the road surface because it is usually less expensive, but for the instances when sea walls are built, sea walls usually protect higher volumes of traffic. While there are few examples of roads that have adapted to HTF (e.g., there are some plans for raising profiles in Florida and New Jersey), our analysis shows that it would be cost-effective for many roads to protect in the next few years, even with an *S*-value of 10, in which protecting 1% of the roads with sea walls protects 20% of the traffic.

**Fig. 7.**
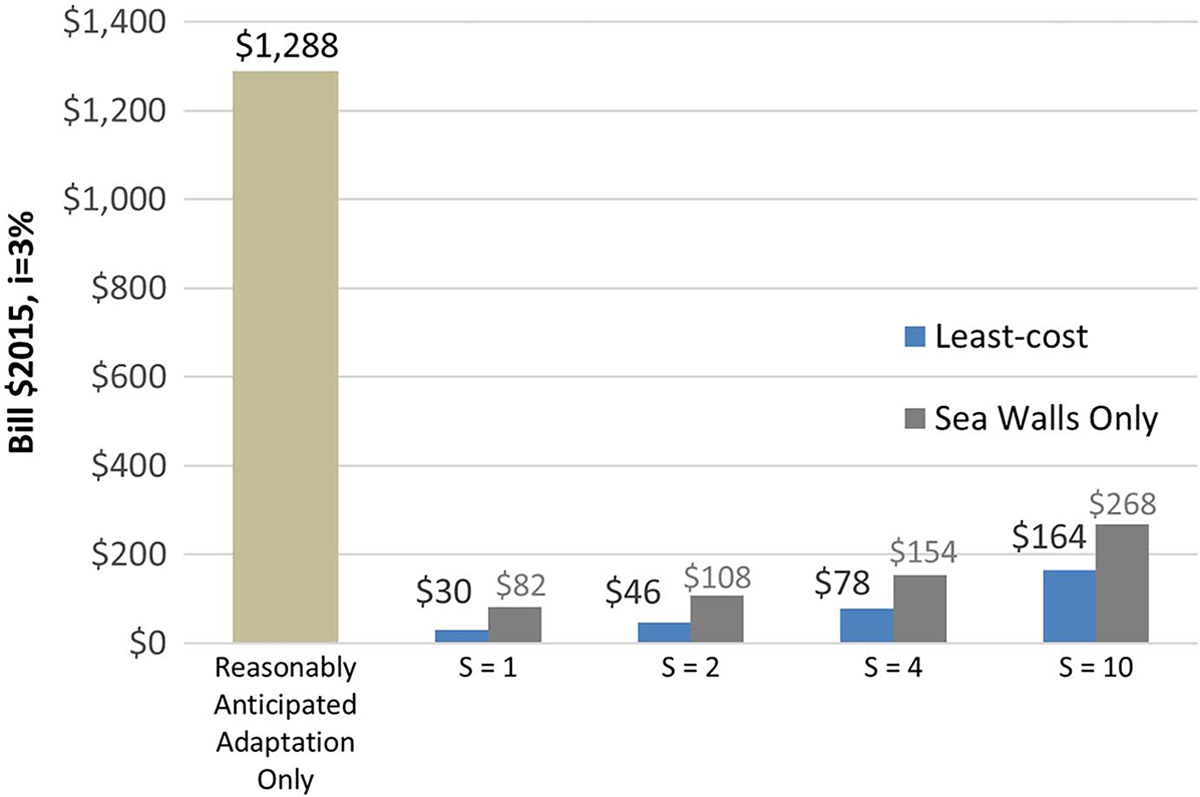
Total costs for RCP 8.5, 2000–2100 (in billions of $2,015) for reasonably anticipated adaptation only and four scenarios with direct adaptation using *S*-values of 1, 2, 4, and 10. The *S*-values indicate the required benefit-cost ratio before action. The right-sided bars and numbers show costs for a scenario with only one adaptation option—sea walls—while the left-sided bars show costs for a scenario with both adaptation options, as described in the section “Alternative Routes,” selecting protection from the least-cost option.

Fig. 7 shows the total discounted costs for the reasonably anticipated adaptation only (i.e., without direct adaptation) and for direct adaptation, varying *S*-values. Costs with direct adaptation are significantly lower, between $30 and $164 billion, or 40 and 8 times lower, across *S*-values. The grey bars show costs for a scenario in which roads, if they are protected, must be protected by sea walls, the more expensive option in most cases. These represent a high-end cost estimate for adaptation and range from $82 to $268 billion across *S*-values.

An *S*-value of four is consistent with some recent research (Multihazard Mitigation Council 2017), which concludes that the benefit-cost ratio of actual flood risk adaptation investments is between 4:1 and 7:1. Finally, Table 3 shows how the total costs vary across the four coastal regions for both RCPs, reasonably anticipated adaptations (i.e., without direct adaptation), and with direct adaptation. Direct adaptation is most effective in the Gulf and least effective in the Pacific, with adaptation effectiveness ratios of 26 and 3.8, respectively, for RCP 8.5. Table S3 shows the 10 counties in which direct adaptation is most effective for RCP 8.5 and an *S*-value of four. Five of these counties are in the Gulf, four are in the Northeast, and one is in the Southeast.

## Discussion

Jacobs et al. (2018a) laid the groundwork for this study by developing a method to identify the HTF risks to traffic. This work has extended Jacobs et al. (2018a) from the East Coast states to the Gulf and Pacific coasts, adding economic valuation, as well as the evaluation of both reasonably anticipated and direct adaptation. HTF may currently be correctly classified as a *nuisance* in many coastal areas of the US, but rising seas will accelerate this threat into a major impact on the coastal road network. National-scale traffic delays are expected to grow steadily over time as sea level rises, but reasonably anticipated adaptation to this threat greatly reduces the impact of HTF events. Nearly half of the impacts occur in the Gulf Coast region, owing to a high percentage of low-lying roads along with subsidence and accelerating sea-level rise. Another 25% occur in the North Atlantic region, owing to a high concentration of affected traffic volume near major coastal cities. Direct damage repair costs for a road segment in South Carolina indicate that repair costs are much less than traffic delay costs; however, these costs are, in a sense, a direct adaptation. If the road is not properly maintained, costs will be higher.

Compared to other CIRA categories of impacts, these costs are significant. In this study, the annual end-century impacts with reasonably anticipated adaptation are estimated to be $280 billion under RCP 8.5 and $220 under RCP 4.5. By comparison, the EPA (2017) indicates that annual costs in 2090 under RCP 8.5 for the coastal property sector are $120 billion [based on the model by Neumann et al. (2015b)], $20 billion for the roads sector [based on the model by Neumann et al. (2015a)], and $8.1 billion for inland flooding [based on the model by Wobus et al. (2017)].

Differences between RCP 4.5 and 8.5 simulations indicate costs are reduced by action to mitigate climate change [in the case of HTF, by roughly 25%, similar to the 22% difference noted by the EPA (2017) for the coastal property sector]. But owing mostly to the already committed levels of SLR this century, the impact of greenhouse gas mitigation on HTF is relatively small, consistent with other coastal impact analyses running through 2100 (Dinan 2017; Neumann et al. 2015b; among others).

While HTFs are fluctuating events, they are frequent and somewhat predictable compared to more extreme flooding events, such as hurricanes or riverine flooding. This allows planners and decision-makers to experience HTF impacts in their communities and to be able to anticipate not only similar but also more frequent impacts in the future. There is likely to be significant technological potential to adapt physically and socially in response to such a large threat. Our simulations with two options for direct adaptation—sea walls and raising the road surface—indicate significant savings compared to costs without direct adaptation: costs are potentially reduced to $4 billion annually in 2090 under the *S* = 4 assumption. Our analysis shows that more roads need to be protected from HTF in the coming years, a challenge that will escalate quickly through the end of the century.

It may be tempting to conclude from our results that cost-effective adaptation, including the rerouting of traffic, the timely raising of roads, and construction of seawalls, can be taken as given, and that as a result, HTF impacts may not be an important concern. In practice, there remain many barriers to implementing cost-effective adaptation in all settings. In a general sense, technological, behavioral, and financial barriers stand in the way of achieving an economically optimal adaptation outcome [see a thorough discussion by Chambwera et al. (2014) and more specific examples for the US coastal zone by Moser et al. 2014]. Our modeling in this work assumes that at least three layers of adaptive response will work efficiently together to support an effective response to HTF vulnerability:

- Alternative routes: Because we have not been able to assess road redundancy at a link level, it is possible we have overestimated the impact of alternative routing to avoid HTF delays. Also, additional costs associated with managing road closures and detours are not considered.
- Ancillary protection: To take full effect, homeowners and communities would need to add on SLR and storm surge risk to protect properties at the right time and location—but Lorie et al. (2020) and other work (e.g., Bakkenson and Mendelsohn 2016; Javeline and Kijewski-Correa 2019) suggest decisions to invest in coastal property protection are suboptimal, and there is also a risk that coastal protection has less than 100% efficacy in protecting adjoining roads.
- Direct adaptation: Raising road profiles and sea walls are modeled as an incremental cost during the *next* major rehabilitation cycle. We know that adding a road raising option to these projects may complicate the permitting and execution of rehabilitation, which may happen on a longer than 20-year time cycle (extending delay time) and may involve further complications, such as raising or protecting nonvehicular infrastructure (e.g., above and below-ground utilities, stoplights, sidewalks). All of these could raise the cost of direct adaptation.

Many of these project-level considerations are currently infeasible to model in our national-scale approach, poorly understood in their implications, or lack data. As a result, we caution against using only those results from our work that assume all these elements of adaptive action will operate on time, in a coordinated fashion, and with enough efficacy to realize all the potential benefits of adapting that we model.

In addition, the adoption of the limited benefit-cost method we use to identify likely locations for cost-effective adaptation is not meant to endorse that approach alone in making local adaptation decisions. Ideally, our results will spur local transport authorities and coastal communities to recognize the potentially growing nature of the problem—and then conduct a more nuanced and broader analysis at the project scale to justify HTF adaptation actions. For example, there are numerous additional benefits of adapting, such as an avoided business disruption, property value loss, and local fiscal impact, which can be considered best in a local context. We recognize that expensive capital adaptations will likely need to be justified by some economic analysis. Moss et al. (2019), for example, notes that financial oversight bodies often require benefit/cost analysis for adaptation investments, and we strongly suggest going beyond just the costs of transport delay in characterizing the benefits of adapting for those analyses. Ideally, HTF requires the type of iterative risk management approach recommended in the Fourth US National Climate Assessment (USGCRP 2018) and associated literature (Moss et al. 2019), which considers and then reconsiders climate risks, social and economic context, and technological options both now and as we learn more in the future as uncertainty in these factors resolves, narrows, or increases.

There are other limitations and caveats to note. While the analysis uses projections to the end of the century, the transportation sector may see significant changes over that time that would make some of our assumptions invalid, such as a transition to driverless vehicles or a significant increase in mass transit. Because we rely on a consistent cost per hour of delay throughout the century, this unit cost is representative of the current transportation sector, which is likely to change in a future system significantly. Without direct adaptation, costs scale linearly with the per hour delay costs, i.e., a 50% increase in per hour delay costs increases national costs by 50%, but costs with direct adaptation have a more nuanced sensitivity to per hour delay cost, in which an increase of 50% increases total century discounted costs (at 3%) to 48% but a decrease in perhour delay cost only reduces total national discounted costs by 6%. This suggests costs with direct adaptation are more sensitive to increases in per-hour delay costs than decreases. We also recognize that the chosen discount rate is important, which reflects the tradeoff between consumption today and consumption tomorrow. This paper uses 3%, a commonly employed rate in the climate impacts literature (e.g., Goulder and Williams 2012). There is more on this in Fig. S7 in the Supplemental Materials.

The analysis is also limited to road segments within the flood extent for the current minor flood level. Sea level rise will expand to this extent, and additional roads will become vulnerable, likely causing more delays and road damage than determined in this study. Based on our approximation, total vulnerable traffic could increase by about half in 2050 and more than double by 2100. Absent of road surface elevations, actual flood depth is uncertain, and vehicles may be able to traverse shallow water with only minor speed reductions. Costs are sensitive to depth by about 0.9%/cm. In addition, these flood events are limited to high tide events only. Additional flooding as a result of rainfall or riverine flooding, which are not estimated in this study, may exacerbate the flood extent and/or flood duration if they occur simultaneously with a high tide event. Underground roads are not considered, where flooding may cause significant damage. This study was limited in the site-specific information available, which constrained our ability to investigate effects, such as direct damage to the road infrastructure, so we discourage the use of these results at the project level. Future research should make use of finer resolution topography data, road conditions, drainage, and traffic/routing information. High-resolution inundation maps at various levels are likely to provide a better approximation of the traffic delay risks. Also, sea level and high tide durations and frequencies vary along the coast (these variations are not captured in this study) and result in a lower accuracy for areas further from tide gauges. For smaller-scale studies, it is essential to identify thresholds of impacts important to road managers and users, which might trigger more aggressive and/or permanent responses to this risk over time (e.g., de facto or planned abandonment of certain high-risk roadways. For example, the authors are aware of situations that have already arisen in some coastal communities that have triggered current decisions to abandon roads that currently experience low levels of HTF effectively, but it is clear that problems will worsen).

There are many direct adaptation options to alleviate HTF impacts. We have only considered two protection methods of the many possible adaptation approaches. For example, hydrologic infrastructure, such as dams or pumps, may be used to alleviate high tide flood events. It may be possible in the near term to build temporary protection or use portable pumps if an HTF event is likely to occur. Furthermore, hard-engineered adaptation options, like those evaluated in this study, may not be the most efficient; for example, a viable option may involve altered city planning to direct traffic away from low-elevation roads. These options should be considered in future research. In addition, a systems modeling approach may indicate interactions and cobenefits across sectors. For example, these include merging models of impacts and adaptation of risks to transport, such as this, with property risk models and other types of infrastructure to understand better interactions among atrisk stocks and flows of economic value and productivity, including potentially changing patterns of locations for working, living, and moving around.

## Supplementary Material

Supplemental Material

## Figures and Tables

**Table 1.** Direct repair and indirect costs for a segment along Route 21N near Hilton Head, SC

Sea level rise scenario	Mean hours inundated/year	Indirect delay costs ($/year)	Additional pavement (total inches by 2050)	Direct repair costs [$/year/0.1 mi (0.16 km) inundated]

Intermediate-low (0.5 m)	108	428,673	0.89 cm (0.35 in.)	3,323
Intermediate (1 m)	303	1,201,766	4.06 (1.60 in.)	15,340

**Table 2.** Portion of total road segments and traffic protected by the direct adaptation for the 100-cm (intermediate) SLR scenario for the four *S*-value scenarios

*S*	Year	Segments protected		Traffic protected	
		Raised profile (%)	Sea walls (%)	Raised profile (%)	Sea walls (%)

*S* = 1	2020	15	3	29	33
	2050	45	4	37	35
	2100	68	4	40	35
*S* = 2	2020	12	3	20	31
	2050	37	4	33	36
	2100	66	4	38	36
*S* = 4	2020	5	2	11	28
	2050	22	4	27	37
	2100	62	4	36	38
*S* = 10	2020	1	1	2	20
	2050	14	3	21	35
	2100	52	4	33	38

**Table 3.** Total regional costs for RCP 8.5 and RCP 4.5, 2000–2100 (in billions of $2,015) for reasonably anticipated adaptation only and with direct adaptation using *S*-value 4

Region	Reasonably anticipated adaptation only		With direct adaptation (*S* = 4)	
	RCP 8.5 ($)	RCP 4.5 ($)	RCP 8.5 ($)	RCP 4.5 ($)

North Atlantic	268	191	25	19
South Atlantic	119	77	8	6
Gulf	857	723	33	28
Pacific	44	27	12	8
Total CONUS	1,288	1,018	78	61

## Data Availability

All data used in this analysis are presented in detail in the Supplemental Materials. Some or all data, models, or code generated or used during the study are available in a repository online (indecon.com/iec-climate-change-high-tide-flooding-traffic/) in accordance with funder data retention policies. Some or all data, models, or code used during the study were provided by a third party. Direct requests for these materials may be made to the provider as indicated in the Acknowledgments.
