# Supplementary material for: Mere Nuisance or Growing Threat? The Physical and Economic Impact of High Tide Flooding on US Road Networks
Source: J Infrastruct Syst. Author manuscript; Available in PMC 2022 Sep 15. (PMC9475394; doi:10.1061/(ASCE)IS.1943-555X.0000652)
Supplement: Supplemental Material [file NIHMS1831902-supplement-Supplemental_Material.zip › Supplemental_Materials_IS.1943-555X.0000652_Fant.pdf]

SUPPLEMENTAL MATERIALS

ASCE *Journal of Infrastructure Systems*

Mere Nuisance or Growing Threat? The Physical and Economic Impact of High Tide Flooding on US Road Networks

Charles Fant, Jennifer M. Jacobs, Paul Chinowsky, William Sweet, Natalie Weiss, Jo E. Sias, Jeremy Martinich, and James E. Neumann

DOI: 10.1061/(ASCE)IS.1943-555X.0000652

© ASCE 2021

www.ascelibrary.org

Data and Sources

The following table shows the data names, descriptions, and sources as well as the code and data repository.

Table S1: Table of data and sources

Name	Description	Data Source(s) and/or EPA Dissemination Plan
**a. Data Used in this Study**		
Infrastructure Roads Data	We used Highway Performance Monitoring System (HPMS) shapefile data from 2016, and where noted in the paper, 2015, for the roads and bridge locations. We processed the roads data for each coastal state based on the methods outlined in Jacobs et al. (2016). Separate analyses were performed for functional classes 1-2 and 3-7.	HPMS shapefile data were downloaded from the HPMS Public Release of Geospatial Data website for each state, https://www.fhwa.dot.gov/policyinformation/hpms/shapefiles.cfm
Traffic and Ridership Data	The HPMS spatial data contained data on the AADT, lane width, and surface type. These data are used in various parts of the analysis, including the traffic delay estimation, the road redundancy calculation, and the direct costs, respectively.	U.S. DOT Federal Highway Administration. 2016. Highway Performance Monitoring System Field Manual. Control No. 2125-0028.
National Coastal Property Model (NCPM)	The National Coastal Property Model was developed in order to estimate coastal flood damages in the U.S. as a result of different sea level rise and storm surge scenarios. The model estimates flood and inundation damages, as well as the cost of investments in coastal protection. The model was used to designate roads protected from actions to protect property (Ancillary Protection).	Neumann, J. E., Hudgens, D. E., Herter, J., & Martinich, J. (2010). Assessing sea-level rise impacts: a GIS-based framework and application to coastal New Jersey. *Coastal management*, 38(4), 433-455.<br><br>Neumann, J. E., Emanuel, K., Ravela, S., Ludwig, L., Kirshen, P., Bosma, K., & Martinich, J. (2015). Joint effects of storm surge and sea-level rise on US Coasts: new economic estimates of impacts, adaptation, and benefits of mitigation policy. *Climatic Change*, 129(1-2), 337-349.<br><br>Lorie., M, et al. (2019, in review). Modeling Coastal Flood Risk and Adaptation Response under Future Climate Conditions. *Climate Risk Management*.
Coastal Flood Exposure Map	Raster datasets containing the extent of the flood at the minor level for each state were provided by NOAA and used to determine which road segments are vulnerable to tidal flooding.	National Ocean Service, Office of Coastal Management (2019). Coastal Flood Exposure Mapper. National Oceanic and Atmospheric Administration. www.coast.noaa.gov/floodexposure/ Accessed May 2019.
Sea Level Rise scenarios	Sea level projections for U.S. coastlines produced for the Fourth National Climate Assessment at a 1 degree spatial resolution and a decadal temporal resolution.	Sweet, W. V., Kopp, R. E., Weaver, C. P., Obeysekera, J., Horton, R. M., Thieler, E. R., & Zervas, C. (2017). Global and regional sea level rise scenarios for the United States. NOAA Technical Report NOS CO-OPS 083.
Tide gauge data	Hourly water levels from tide gauge stations were obtained from NOAA's Center for Operational Oceanographic Products and	Sweet, William, Greg Dusek, Jayantha Obeysekera, John J. Marra (2018) Patterns and Projections of

	Services (NOS 2019) and methods for analysis are described in Sweet et al. (2018). 83 tide gauges from this set are in CONUS and we use 19-years of hourly water levels spanning from 1999 to 2017.	High Tide Flooding Along the U.S. Coastline Using a Common Impact Threshold. NOAA Technical Report NOS CO-OPS 086.
Welfare Economic Cost of Traffic Delays	Different sources were used for passenger and freight vehicles. For passenger vehicles, the approach follows that recommended in U.S. DOT (2016b)<br><br>To quantify the cost of delay for freight vehicle travel, we rely on data from the National Cooperative Highway Research Program (NCHRP) that are used as inputs to their Truck Freight Reliability Valuation Model (NCHRP 2016).	Passenger vehicles: U.S. DOT. 2016b. *Revised Departmental Guidance on Valuation of Travel Time in Economic Analysis*. Downloaded from https://www.transportation.gov/sites/dot.gov/files/docs/2016%20Revised%20Value%20of%20Travel%20Time%20Guidance.pdf<br><br>Freight vehicles: Methodology for Estimating the Value of Travel Time Reliability for Truck Freight System Users, document available along with Excel-based Truck Freight Reliability Valuation Model and User's Guide at: http://www.trb.org/Publications/Blurbs/174297.aspx
Direct Costs to Road Surface	All data used in the analysis on direct damages through road maintenance costs are available in the HPMS shape file dataset. Data from 2016 was used primarily, except for fields "Base_type," "Base_thickness," and "Thickness," which were only available in the 2015 HPMS data.	HPMS shapefile data were downloaded from the HPMS Public Release of Geospatial Data website for each state, https://www.fhwa.dot.gov/policyinformation/hpms/shapefiles.cfm
**b. Data Produced by This Study**		
Full results and analysis code	Shape file of the processed road segments within the flood plain for all coastal counties.	Data will be posted to ScienceHub and EPA's Environmental Dataset Gateway (EDG) upon publication of the manuscript.
	Python code for processing the raw HPMS data into processed segments and identifying the segments within the flood plain.	
	Delays and Costs by county, year, sea level rise scenario, and adaptation scenario.	
	Matlab code for road data statistics, distillation of the processed road segment dataset, and estimation of delays and costs for all scenarios.	

Road and traffic processing details

For functional classes 1 and 2, HPMS data for both directions of the highway are typically combined and reported for only one direction of the highway; we distinguished the "non-inventory" direction of the highway using the facility type attribute (HPMS 2016). All states, except for New York and New Jersey, use this reporting style in their HPMS data. We only process the inventory side of the highways for the analysis, which inherently assumes that if the inventory side experiences HTF, then the non-inventory direction will also flood. Figure S1d shows an example where the red line shows the side of the road, which appears in the HPMS dataset and is evaluated in this study while the other side is visible is missing in the HPMS dataset but appears in the satellite imagery.

We identified multiple states where HPMS-identified bridges did not align directly with satellite imagery of the roads. In effect, the foot of the bridges intersected the two-dimensional flood plain in the model causing elevated bridges in the model to be flagged as flooded. To address these false positives, we removed the bridge plus a 200-ft buffer surrounding the bridge from the roadways prior to calculating flooded segments. The 200-ft buffer fixes the vast majority of these errors, but preserves most situations where the segment is flooded near the base of the bridge (avoiding false negatives). Figure S1b shows an example of a case with the correct bridge location and Figure S1d shows an example of an incorrect bridge location with the 200 ft buffer correction. The road segments on either side of Figure S1b are not considered flooded once the bridge is removed because the bridge was correctly identified. In contrast, the northern segment in Figure S1d would have been considered flooded without the 200 ft buffer (dashed red line).

Figure S1: Examples of road segmentation process: (a) shows three flooded road segments; (b) a correct bridge location; (c) shifts in functional class; and (d) an incorrect bridge location with the 200 ft buffer correction. (a), (b), and (d) are in Connecticut and (c) is in Texas. (Map data from Esri, Maxar, GeoEye, Earthstar Geographics, CNES/Airbus DS, USDA, USGS, AeroGRID, IGN, and the GIS User Community.)

a) Three Flooded Road Segments

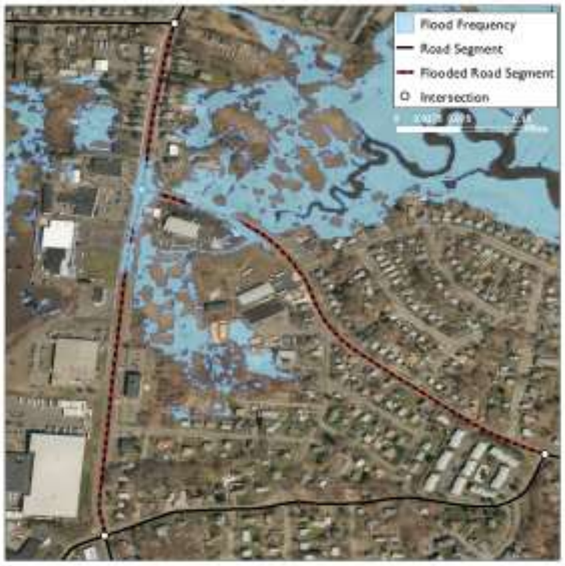

b) Correct Bridge Location

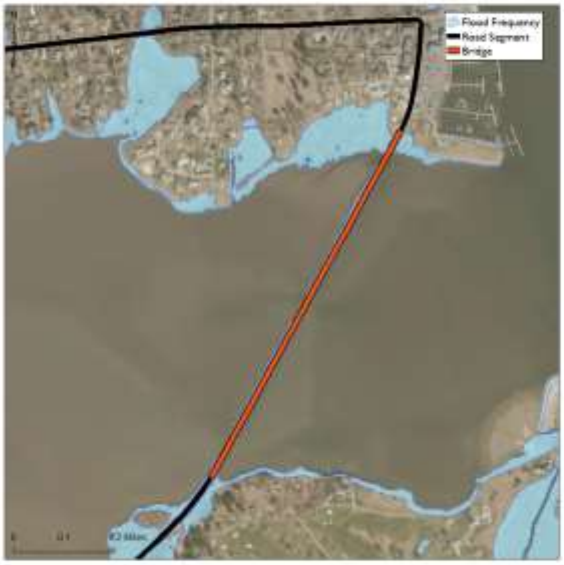

c) Alternating functional class

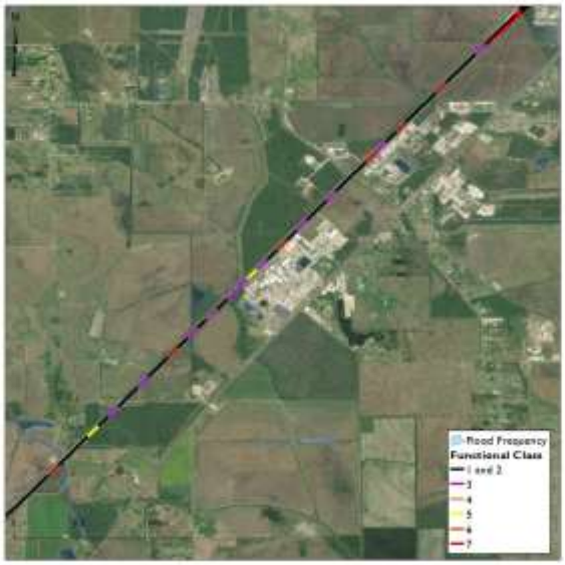

d) Bridge Location Error with Bridge Buffer

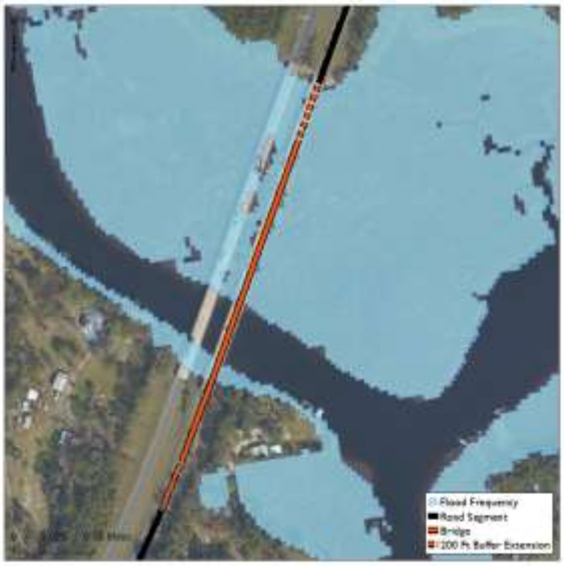

There were a few states that required additional processing. California's 2016 HPMS data were not as complete as the 2015 reporting. Georgia's and Louisiana's 2016 HPMS data did not include ramp

locations. For these three states, we used 2015 HPMS data instead of 2016 HPMS data. Additionally, Georgia's 2015 and 2016 data did not report consistent bridge locations, so we merged bridge segments from both years and used this set to determine bridge locations in the analysis. Some roads in the Texas and Virginia HPMS alternate between functional classes between intersections (see Figure S1c). Because our approach processes Functional 1 and 2 roads independent of functional classes 3-7, these two functional class groups are disconnected, which results in gaps in the road for both functional class groups (where the other group would fill the gap). In some cases, the flood plain may overlap with the road within these gaps. No additional steps were taken to rectify this result, which contributes to the overall conservative nature of the assessment – that is, effects are likely underestimated for these states.

Ramp segments are ignored in the delay calculations, consistent with Jacobs et al. (2018). Because many states do not report AADT data for functional classes 6 and 7 roads, we imputed AADT values for these roads. We assume that if the road's AADT is 0, data are missing. If the state reports AADT values for more than 50% of functional class 6 and 7 roads then the state average AADT value for functional class 6 and 7 roads, respectively, is assigned where data are unavailable. If the state reports AADT values for less than 50% of functional class 6 and 7 roads' AADT, we used the average AADT from all the coastal states for functional class 6 and 7 roads, respectively, for missing values.

Road Maintenance Costs

In coastal regions where extreme weather and high tide flooding routinely inundate roads, premature pavement failure is likely to occur for asphalt concrete (AC) pavements, but is much less likely to occur for Portland Cement Concrete (PCC) pavements. Damage to flooded pavements results because the unbound pavement layers in saturated pavements are weakened and the pavement structure has a reduced load-bearing capacity. Limited studies have shown that a 25% reduction in load-bearing capacity is a typical value (Zhang et al. 2008, Vennapusa et al., 2013). However, the vulnerability of a pavement to damage from traffic during flooding depends on several conditions including flood characteristics, pavement materials and thickness, drainage conditions, and surface cracking (Elsaher et al. 2017, Mallick et al. 2017). Additionally, unlike damage to other infrastructure that depends on the depth of inundation, inundation duration rather than depth is more critical for quantifying the damage to roadway.

Ultimately, vehicles, particularly heavy trucks, are the source of pavement damage. Thus, traffic management plays an important role in the amount of damage that a flooded roadway incurs. Agencies that prioritize connectivity and open roads despite reduced capacity may incur more significant repair costs than agencies that choose to limit damage by closing the road until it regains its bearing capacity.

Any specific section of road may be studied in detail to evaluate the potential for additional damage induced on a flooded pavement structure given site-specific conditions. The approach employed in this study is to calculate the reduction in pavement life using the AASHTO 1993 pavement design equation, which accounts for pavement structure, traffic, and layer moduli. The increase in damage due to traffic passing over a weakened pavement structure is calculated by using saturated moduli values for the unbound pavement layers during the recovery period. The recovery period is estimated from hydraulic modelling of pavement structures to determine the time to inundation and time for recovery (unbound

materials return to normal moisture conditions). The Equivalent Single Axle Load (ESAL) can be estimated from a traffic model, which considers Annual Average Daily Traffic (AADT), truck percentage, vehicle class distribution and load equivalency factors (for interstate or primary roads) to ESAL. The additional thickness of asphalt surface material needed to carry the traffic without additional damage is then determined. The cost is then calculated by determining the volume of material needed (multiplying thickness by lane width and segment length) and multiplying by in-place cost.

To understand the impacts at a regional or national scale, a potential approach is to use the methods described the primary manuscript to quantify the damage due to flooding for a given flood duration for a range of conditions (see the criteria below) as needed to develop look-up tables that are broadly applicable. These scaled damage values could then be applied to specific road segments inundation duration to determine the segment damage and, ultimately, cost due to damage for an individual segment. The following six criteria would likely require approximately 100 (or more) combinations to be considered using layered elastic analysis under flood and normal conditions. The total number of combinations depends on the level of detail required for each criterion. The criteria are listed below.

Hydrology and hydraulic conditions

Unfavorable hydrological conditions e.g. river flooding can keep the subgrade weak over a long period. In areas where the existing groundwater level is high before flooding, pavement resistance to rutting can be more vulnerable to the additional moisture and moisture may be more difficult to drain away. Usually, repeat offenders have poor combinations of hydrological and hydraulic conditions (e.g., close to a body of water, wetlands, or subject to thaw in spring). These pavements are susceptible for high moisture content in the structure, even without being flooded, and the recovery period is usually longer. Hydrologic conditions using national soil survey and wetlands information could be considered to be poor or okay.

Drainage conditions

Similar to hydrologic conditions, a reduction in stiffness is expected when unbound (crushed gravel) and subgrade (sandy silt) materials are partially/fully saturated. Insufficient drainage such limited elevation from the ground to the pavement section or due to blocked ditch and subdrainage (e.g., fillers and pipes) can extend the recovery period. Drainage conditions could be considered to be poor or okay based on slope, elevation or adjacent conditions.

Unbound and subgrade materials

Pavement with silt or clay subgrade may be more vulnerable to post-flooding damage. Silty and clay material, once saturated, takes longer to recover due to the poor permeability, compared to gravel or sandy soil. With a longer recovery period the post-flooding road has more exposure to loss of stiffness and damage under traffic. Additionally, silty or clay soil may have less in-situ stiffness compared to gravel or sandy soil, and can have a greater percentage of stiffness reduction due to full saturation. Subgrade conditions using national soil survey and wetlands information could be considered to be vulnerable or not vulnerable.

Structure

Thin pavements usually have less ability to spread traffic loading and the critical stress and strain can be greater compared to thick pavements in normal and inundated conditions. While there is no strict threshold to distinguish between thick and thin pavement, a pavement could be considered to be thin or thick based on its functional classification or FHWA databases.

Surface cracking

Some pavements are designed with impermeable surface to prevent water ingress. However, flooding water and rainfall can enter the pavements through cracking. Depending on the severity of the cracking (area, length, width and depth), the flow of surface water into pavements can vary and this needs to be

classified. Surface cracking information is available from FHWA databases at a range of details and likely could be classified as low, medium, or high.

Traffic

Traffic is another important factor to consider for the vulnerability of post-flooding roads, as traffic loading is the source of damage and is assumed to be dependent on the volume of heavy truck traffic, if the heavy truck traffic is below an established threshold. This is because that the damage is mainly caused by the heavy trucks. If the truck traffic volume is above a certain threshold, no additional impacts occur regardless of how much more traffic it is above the threshold. Traffic information is available from FHWA databases.

Alternative Routes Approach

The relative traffic intensity, R, is used to reduce total calculated delays as a quantitative indicator of driver-initiated delay reductions. The relative traffic intensity, R, calculated as

$$R = \frac{\sum(AADT * L)}{\sum(Cap * L) * 24hrs} \quad \text{[Eq S1]}$$

where L is road segment length and Cap is the road capacity in vehicles per hour, which excludes the capacity of the flooded segment. The AADT and capacity are summed, length-weighted, for all segments in a consistent specified area. For this search area, we use half of the average trip length in the U.S. of roughly 10 miles (DOT 2018[1]) as the search radius along the road. For example, an R value of 0.1 indicates there is low traffic to capacity ratio in the search radius with high potential to reroute, decreasing delays by 90%, while a value of 0.6 indicates low potential to reroute and only decreases delays by 40%.

Road capacity is the “maximum reasonable hourly rate at which vehicles can be expected to transverse a point or a uniform section of lane or roadway during a given time period under prevailing roadway, traffic, and control conditions” (HPMS 2016). Road capacity is determined from the HPMS data, to the extent possible (USDOT 2017), which uses a combination of the Highway Economic Requirements System (HRES) capacity procedure (HERS 2014) and the National Cooperative Highway Research Program (NCHRP; Dowling et al. 2016). Each of the six road categories, freeway, stop-controlled highways, signalized highways, multilane highways, rural highways, and urban highways, follows a different set of equations to estimate capacity, all of which are provided in USDOT (2017). Default values are used when the information is missing from the HPMS database (USDOT 2017). Note that the road categories used here are not the same as the HPMS functional classes used in the road segmentation.

---

[1] U.S. Department of Transportation, Bureau of Transportation Statistics, Federal Highway Administration, National Household Travel Survey data, May 18, 2018.

Direct Adaptation Costs

The cost of adaptation, C, varies by adaptation type. The cost of the sea wall is

$$C_{sw} = K_{sw} L H^2 (1 + M_{sw}) \quad \text{[Eq S2]}$$

where K_{sw} is the unit capital cost for a sea wall that is 1 m high and 1 m long; L is the length of along the road that is in the flood plain in m; H is the height of sea wall required to protect the segment up to the end of the expected life of the sea wall in m; and M_{sw} is the operation and maintenance cost expressed as a fraction of the capital cost summed over the planning horizon, discounted by 3%. For the unit capital cost, we use $4,513 per linear meter, expressed in 2015 dollars (Neumann et al. 2015a) and is consistent with the costs for ancillary protection. The life of the sea wall is assumed to be 50 years. The height of the sea wall is the increase of the HTF threshold with sea level rise for the sea level rise projection scenario (e.g., 50 cm or 100 cm) at the end of the expected life. For the operation and maintenance cost, we assume 3% of capital costs per year, as used in Neumann et al. (2015a). The regions where sea walls cannot effectively prevent flooding due to deep porous soils were not identified. We also assume that building a sea wall at a height less than 0.5 m would be infeasible, so this is used as a minimum height in the cost estimation. Similarly, we assume the length of protection along the road will be at least 0.5 miles, which applies to both sea walls and raising the road profile.

A road profile is raised by increasing the base thickness of an existing road. Here, we assume that the base layer is increased during a regular maintenance cycle so that costs associated with replacing the asphalt layer are excluded because those costs are incurred whether or not the road profile is raised in response to HTF. The cost for raising the road profile (C_{rp}) is calculated as

$$C_{rp} = L\left[(BHNW + EH^2)\left(1 + M_{rp}\right) + DC_{delay}HNW\right] \quad \text{[Eq 5]}$$

where B is the cost of adding base thickness to the road per lane mile per ft raised per ft of lane width; N is the number of lanes; W is the lane width; E is the embankment cost per square foot per mile length; M_{rp} is the operation and maintenance cost for raising the profile expressed as a fraction of the capital cost summed over the planning horizon; D is the construction delay in hours per ft width per ft raised per mile length; and C_{delay} is the delay cost per hour, which depends on passenger and heavy vehicle traffic. Labor costs are built into the material costs for the base and embankment costs. Most of the unit costs are derived from the RSMeans software (Guardians 2019). However, for the base costs we use an average of RSmeans and the cost used in Knott et al. (2019), which comes to $125,000 per lane mile per ft raised for a 13 ft lane. The number of lanes and lane width are road-specific and available through the HPMS roads dataset. When these are missing from HPMS, we use the state-wide average lane width with two lanes. We assume an earthen embankment on both sides of the road is built at a 45° angle with a cost of $9 per cubic yard. Incremental construction delays are 6 days per ft raised for a 13 ft lane (Guardians 2019). We use a maintenance cycle of 20 years. In most cases, the first time the profile is raised for a road will not occur on a regular maintenance year. To account for the disparity, we delay raising the profile by half the maintenance cycle, 10 years, from the year the B/C threshold is reached.

In heavily developed areas, raising the profile may be problematic and unreasonably expensive because of the connections to the road and/or obstacles that restrict the additional width required. The HPMS dataset includes a field for the presence of a widening obstacle but only about 10% of the roads include this information. Instead of costing these location-specific complications, we flag a road as not suitable for raising the profile when either there is a widening obstacle or when the road is four lanes and designated with an urban code. Acknowledging that this assumption may introduce error, we run a sensitivity scenario that requires that direct adaptation uses only sea walls, even when raising the profile is the cheaper option.

Table S2: Summary table of what is included and excluded in direct adaptation costs for the two direct adaptation options: raising the road profile and sea walls

RAISING ROAD PROFILE	
**Costs include**	**Costs do not include**
• Materials (RS Means)<br>• Labor (RS Means)<br>• Delay costs during construction	• Construction management (provided project is part of period rehabilitation)<br>• Rebuilding driveways<br>• Rebuilding sidewalks<br>• Complications near intersections<br>• Rebuilding or moving utility infrastructure such as utility poles or pad-mounted transformers<br>• Maintaining access to underground infrastructure such as water, sewer, buried electrical or ICT<br>• Managing issues with overhead clearance, such as overpasses
**SEA WALLS**	
**Costs include**	**Costs do not include**
• Materials (Herberger et al. 2009)<br>• Labor (Herberger et al. 2009)	• Pumping system to account for groundwater intrusion<br>• Land Acquisition, if necessary<br>• Land clearing or site leveling<br>• Access roadways for maintenance<br>Aesthetic or usefulness improvements

Additional Results

Figure S2: State total mean daily traffic at risk, i.e., sum of traffic from all road segments in the high tide flood plain, sorted from highest on the left to lowest on the right. The black line shows the cumulative vulnerable traffic and corresponds to the right vertical axis.

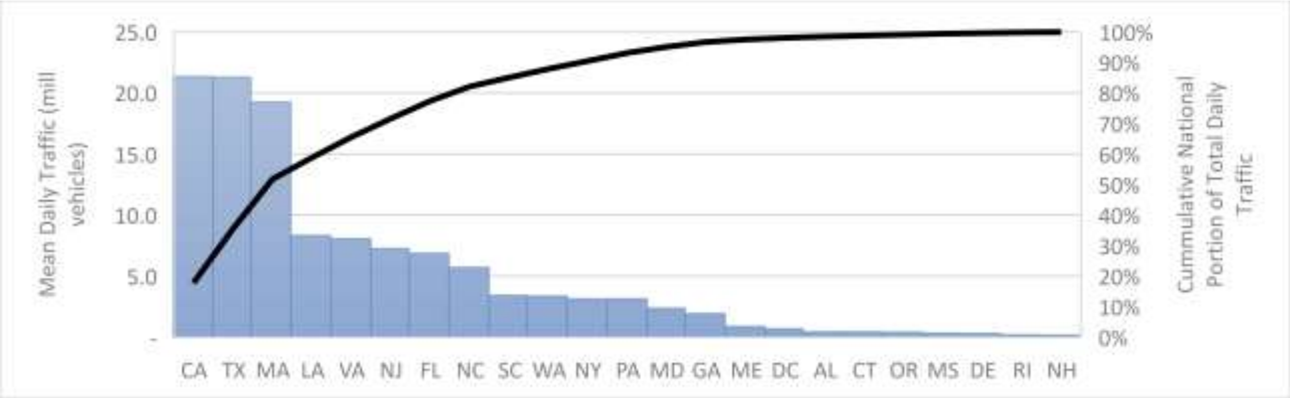

To understand the impact of the effective threshold, we developed a simple sensitivity analysis using 10 cm lower, 10 cm higher, and 20 cm higher than the minor level. The results of this are shown in Figure S3. As shown, increasing or decreasing the effective depth shifts delay results by about 0.9% per cm.

Figure S3: Delays in CONUS for four effective thresholds relative to the minor level for RCP 8.5

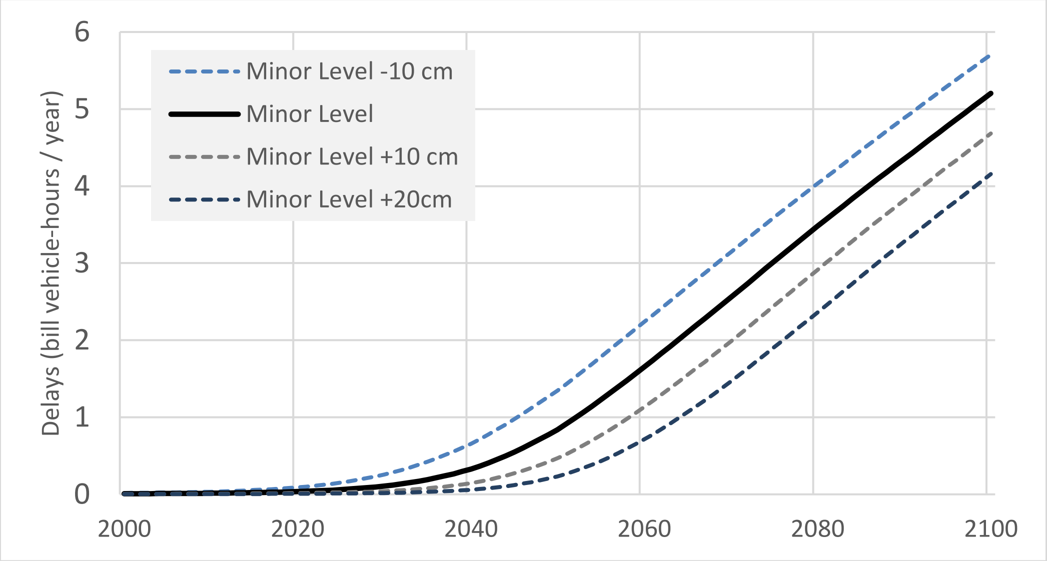

Figure S4: Map of delay costs (in millions of 2015 USD / year) by county for reasonably anticipated adaptation, RCP4.5 and RCP 8.5

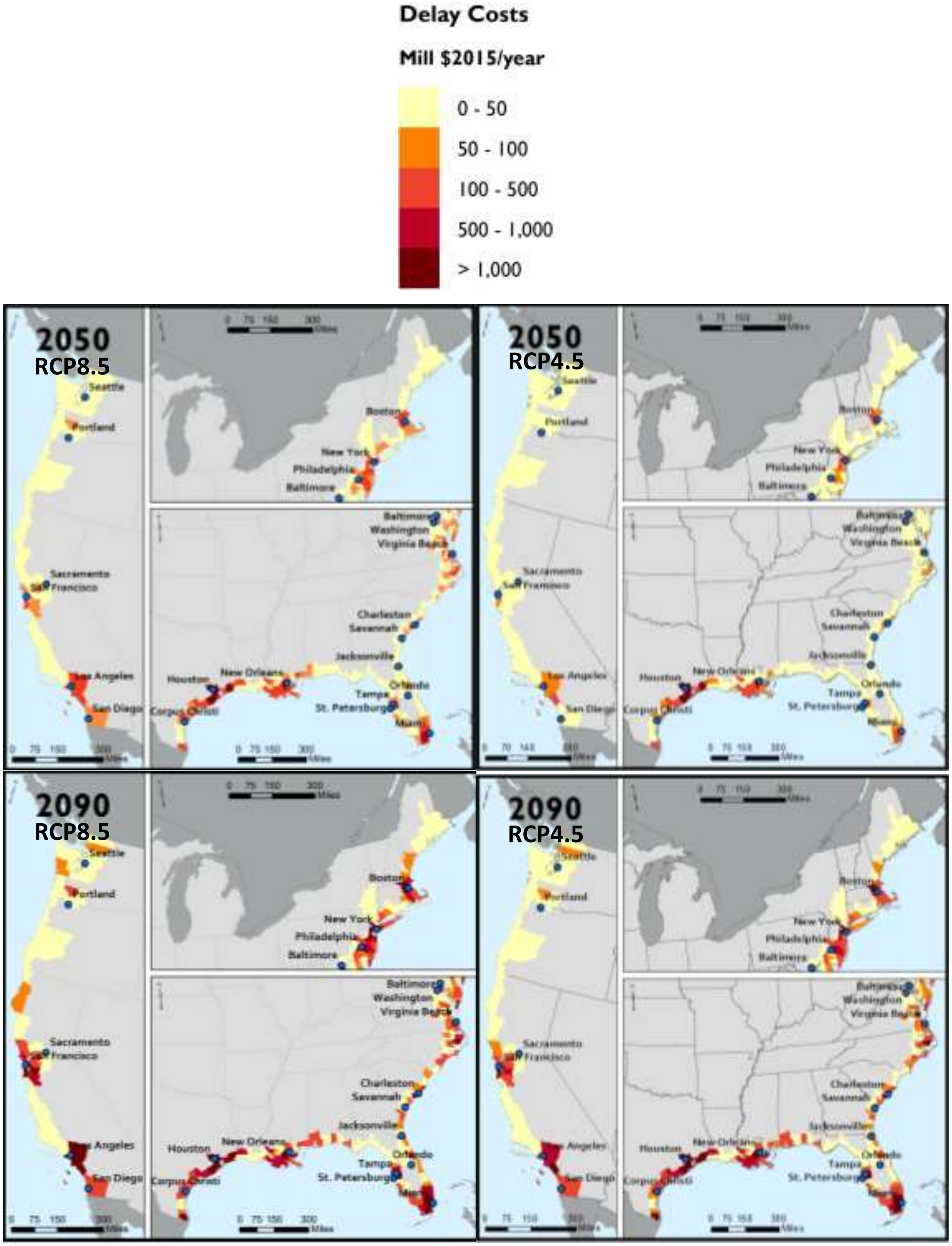

Table S3 shows the counties where direct adaptation is most effective for RCP 8.5 and an S-value of four. Four of the top five counties are in the Northeast around New York City. These roads are often heavily trafficked, which allows for projects to efficiently reduce traffic delays. In contrast, Orange County, California, where total cost without direct adaptation is $8.9 billion, has an adaptation ratio of 3 due to more road segments with lower traffic. Other notable areas are Richmond, VA, Tampa, FL, and New Orleans, LA, all of which are low-lying cities in the southeast where sea level rise is expected to accelerate quickly.

Table S3: Counties where adaptation is most effective (results for S=4, RCP 8.5), total discounted costs for 2000-2001 and adaptation ratios: without direct adaptation over with direct adaptation.

Rank	County (Largest city in or near county)	Without Direct Adaptation ($mill)	With Direct Adaptation ($Mill)	Adaptation Ratio (without / with adapt)
1	Union, NJ (Elizabeth, NJ)	$12,373	$18	681
2	Kings, NY (Brooklyn, NY)	$3,335	$6	582
3	Iberville, LA (south of Baton Rouge)	$5,649	$30	189
4	Middlesex, NJ (Edison, NJ)	$7,307	$64	114
5	Bronx, NY (New York, NY)	$10,041	$96	104
6	Miami Dade, FL (Miami, FL)	$60,465	$861	70
7	Richmond City, VA (Richmond, VA)	$2,041	$38	54
8	Hillsborough, FL (Tampa, FL)	$6,700	$131	51
9	Orleans, LA (New Orleans, LA)	$267,472	$5,393	50
10	Jefferson, LA (West of New Orleans, LA)	$92,372	$2,062	45

Table S4 summarizes the annual costs for each adaptation scenario and 6 regions for 20-year eras (2030: 2020-2039, 2050: 2040-2059, 2070: 2060-2079, 2090:2080-2099). These regions are the same as the 4 regions used in the manuscript except that the Pacific and Gulf are split into two regions each that correspond with the regions used in the Fourth National Climate Assessment (NCA).

Table S4: Annual costs for each region and adaptation scenario

	Delay Costs with no adaptation ($Mill USD/year)							
	RCP 8.5				RCP 4.5			
NCA Region	2030	2050	2070	2100	2030	2050	2070	2090
Northeast	$5,413	$21,261	$67,206	$141,648	$7,740	$39,171	$133,988	$275,633
Southeast - Atlantic	$954	$5,424	$22,171	$54,752	$1,479	$10,916	$49,449	$118,955
Southeast - Gulf	$4,592	$39,270	$123,481	$210,643	$6,597	$58,321	$161,466	$248,118
Southern Great Plains	$9,398	$74,867	$259,292	$490,314	$13,984	$122,411	$371,124	$592,771
Southwest	$393	$1,924	$7,897	$23,472	$550	$3,588	$17,662	$57,441
Northwest	$186	$371	$845	$2,040	$213	$535	$1,586	$4,651
TOTAL	$20,936	$143,117	$480,892	$922,869	$30,563	$234,942	$735,274	$1,297,570
	Delay Costs with reasonably anticipated adaptation ($Mill USD/year)							
	RCP 8.5				RCP 4.5			
NCA Region	2030	2050	2070	2100	2030	2050	2070	2090
Northeast	$959	$3,624	$11,123	$23,323	$1,367	$6,528	$21,934	$45,672
Southeast - Atlantic	$128	$885	$4,242	$11,231	$208	$1,886	$10,036	$25,771
Southeast - Gulf	$1,353	$10,031	$31,385	$54,998	$1,887	$14,856	$41,830	$65,173
Southern Great Plains	$1,049	$8,034	$27,766	$52,731	$1,546	$13,108	$39,862	$64,087
Southwest	$61	$301	$1,246	$3,727	$85	$563	$2,795	$9,133
Northwest	$17	$34	$79	$189	$19	$50	$147	$429
TOTAL	$3,567	$22,909	$75,840	$146,198	$5,112	$36,991	$116,604	$210,265
	Delay Costs with direct adaptation ($Mill USD/year)							
	RCP 8.5				RCP 4.5			
NCA Region	2030	2050	2070	2100	2030	2050	2070	2090
Northeast	$339	$544	$578	$625	$518	$915	$950	$1,112
Southeast - Atlantic	$87	$217	$272	$328	$140	$370	$443	$548
Southeast - Gulf	$211	$180	$128	$182	$298	$257	$180	$293
Southern Great Plains	$564	$845	$418	$380	$794	$1,013	$481	$591
Southwest	$57	$200	$401	$539	$81	$378	$812	$977
Northwest	$14	$30	$59	$101	$18	$46	$112	$215
TOTAL	$1,273	$2,015	$1,855	$2,154	$1,849	$2,979	$2,978	$3,736
